# Chemical and isotopic characterization of the thermal fluids emerging along the North–Northeastern Greece

**DOI:** 10.1038/s41598-021-95656-6

**Published:** 2021-08-11

**Authors:** E. Dotsika, P. Dalampakis, E. Spyridonos, G. Diamantopoulos, P. Karalis, M. Tassi, B. Raco, A. Arvanitis, N. Kolios, J. L. Michelot

**Affiliations:** 1 Stable Isotope Unit, N.C.S.R. “Demokritos”, 15310 Ag. Paraskevi, Attikis, Greece; 2 Institute of Geosciences and Earth Resources, C.N.R., Via G. Moruzzi 1, 56124 Pisa, Italy; 3 Hellenic Agricultural Organization - Demeter, Soil and Water Resources Institute, Sindos, Greece; 4 PPC Renewables S.A, 15343 Ag. Paraskevi, Attikis, Greece; 5 Hellenic Survey of Geology and Mineral Exploration GR, Acharnes, Greece; 6 Institute of Geology and Mineral Exploration (IGME), Thessaloníki, Greece; 7 CNRS, Géosciences Paris Saclay (GEOPS), Université Paris Saclay, Orsay, France

**Keywords:** Geochemistry, Geology

## Abstract

Hydrochemical and isotopic characteristics of fluids from major geothermal fields of middle/low temperature in N/NE Greece are examined [basins: Strymon River (SR), Nestos River Delta (ND), Xanthi–Komotini (XK), Loutros–Feres–Soufli (LFS) and Rhodope Massif]. The geodynamic context is reflected to isotopic/chemical composition of fluids, heat flow values and elevated CO_2_ concentrations in emitted fluids. B and Li are derived from leaching of the geothermal systems hosting rocks. δ^18^O_H2O_, δ^18^O_SO4_, δ^13^C_CO2_ values and chemical compositions of Cl, B and Li of geothermal discharges suggest two distinct source fluids. Fluids in SR exhibit high B/Cl and Li/Cl ratios, suggesting these constituents are derived from associated magmas of intermediate composition (andesitic rocks). Geothermal discharges in LFS exhibit low B/Cl and Li/Cl ratios, implying acid (rhyolitic) magmatism. δ^13^C_CO2_ and CO_2_/(CO_2_ + 10^5^He) ratios in the west part, suggest fluids affected by addition of volatiles released from subducted marine sediments. For the eastern systems, these ratios suggest gas encountered in systems issued from mixing of crustal and mantle-derived volatiles. Isotopic geothermometers reflect, for the same direction, equilibrium processes more (*LFS*, *XK*) or less (SR) pronounced and discriminate the geothermal field from low to middle [SR, ND (Erasmio)] and middle to high enthalpy [ND (Eratino), LFS, XK].

## Introduction

Isotope geochemistry, has greatly contributed in understanding geothermal systems since chemical and isotopic composition of geothermal fluids provides information on their origin, recharge areas, flow patterns, water–rock interaction processes and geothermal reservoir’s deep temperature^1,2^.

**Figure 1:**
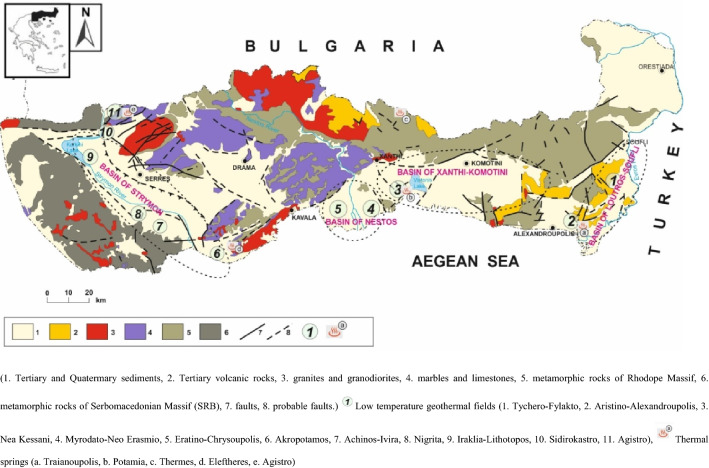
Tertiary sedimentary basins and investigated low temperature geothermal fields located on a simplified geotectonic map of North Eastern Greece (scale 1:500.000^4^).

Geothermal systems with low to high enthalpy are present in North and Northeastern Greece. The wider region (Fig. 1) is extending between the Strymon River in the West and the Evros River in the East (Greek–Turkish borders). The hydrothermal area include Traianoupolis, Fylakto–Tychero [Loutros–Feres–Soufli basin, (LSB)], Nea kessani [Xanthi–Komotini basin (XK)], Erasmio and Eratino [Nestos Delta basin (ND)] and Nigrita–Therma, Sidirokastro, Iraklia, Agistro, Achinos–Ivira and Akropotamos [Strymon River basin (SR)] thermal springs. These thermal waters have been well-known since antiquity. Today, they are mainly used for green-house heating and hydrotherapy. The temperature of these water springs, which present low to high-salinity, ranges from 30° to 80 °C. Please refer to Supplemental material for more information of the study areas.

The thermal exploration of the area began in the 1970’s with an investigation carried out by I.G.M.E. (Institute of Geology and Mineral Exploration). During this stage, the well of Fylakto–Tychero area produces Na–Cl type geothermal waters with minimum flow rate of 200 m^3^/h and temperature of 37.0 °C, while water temperatures up to 99 °C and flow rates higher than 100 m^3^/h have been measured during wells production tests in the Aristino–Traianoupoli area^3^. The eastern and central part of the Xanthi–Komotini basin is characterized by a relatively elevated heat flow regime and the subsequent presence of geothermal aquifers with low water temperatures ranging between 30 and 45 °C at depths 400–500 m. On the contrary, on the western margin of the basin and most precisely in the area of Nea Kessani–Xanthi, the main hot reservoir is found at shallow depths (150–400 m) with water temperatures ranging from 75 to 82 °C^4^. In Nestos Delta basin the maximum depth of the 22 exploration boreholes drilled by I.G.M.E. reaches 500 m. The values of the geothermal gradient are very high (up to 25 °C/100 m). The water temperatures range from 40 to 72 °C. The geothermal gradient for the Strymon basin has been estimated to fluctuate from 25 to 36 °C/km at depths over than 2000 m^3^.

The geochemical characteristics of these thermal waters have already been investigated in few studies. Especially, the chemistry of thermal springs has been investigated in various studies^5–7^, and mainly at Kollios et al.^4^ and are focused on in the N. Kessani–Evros area. Dotsika et al.^8,9^ elucidated also the isotopic characteristics of thermal water discharges in the Northern and Northern Eastern Greece. The comprehensive investigation by Dotsika et al.^8,9^ provided also, except from chemical and isotopic data (δ^2^H and δ^18^O of H_2_O, δ^34^S) the application of isotopic geothermometers for these thermal waters.

Therefore, until now, no systematic chemistry and isotopic research has been undertaken on these geothermal waters, low to high enthalpy, which emerge from coastal to volcanic rocks, in order to define the geothermal model of this region. In high enthalpy geothermal systems, the dominated theory for the origin of water was that the thermal fluids were of magmatic and/or juvenile origin and not of meteoric origin. However, through the measurements of the stable isotopes of water and steam of well-known geothermal fields, was proven (Craig, 1963^10^) that the origin of thermal waters could be to be of meteoric origin. The positive shift of δ^18^O of thermal water of meteoric origin is attributed to ^18^O exchange between the water and the reservoir rock's, showing the high temperature of this thermal environment. However, for many water types in Greece, a shift not only in δ^18^O^1^ but also in δD^2,11^ is observed, indicating mixing of local groundwater with marine or magmatic or metamorphic water. The high mobile constituent chloride, which are typically found in most deep aquifers, especially in Na-Cl type waters, are generally attributed to direct seawater intrusion, particularly near the coastal zones and modified seawater.

Additionally, the correlations among δ^18^O, δ^2^H, δ^13^C and mobile specie are particularly important tools used in geochemical investigations, both to detect processes of liquid–vapor separation, absorption of magmatic gases in groundwater, mixing processes and to provide information on water origins. Especially, the δ^18^O–δ^2^H isotope composition of cold ground waters is mainly affected by recharge altitude and mixing of different fluids, while in deep aquifers, isotopic values are governed by the water–rock interaction, mixing and boiling (vapor separation). The δ^13^C and δ^34^S values of dissolved inorganic carbon and sulphate in thermal and cold waters are used to identify the provenance of CO_2_, sulphate participation of marine origin, mixing of sulfates of ‘different’ origin (marine sulfate, sulfates resulting from sulfur oxidation and from dissolution of sulfur of deep rocks).

In this study the stable isotopes of water (δ^2^H, δ^18^O), carbonate (δ^13^C), sulfate (δ^34^S, δ^18^O) values with the chemical solutes, major and minor (Br, Li, B) ions, are used in order to identify the possible origin of thermal water as well as the possible procedures responsible (ex. water–rock interaction processes) for the alteration of the initial composition of these waters and to allows one to reconstruct the conceptual geochemical model of the investigated area. The use of isotopic and chemical geothermometers will provide also an estimation of the temperature of the deep geothermal fluids.

### Geological framework

The geological evolution of North Eastern Greece is related to the Late Cretaceous closure of the Vardar Ocean when subduction related magmatism developed in the Eastern European margin causing the consumption of different portions of Tethys Ocean (e.g., Stampfli and Borel 2004)^12^. It belongs mainly to the crystalline mass of the Rhodope Massif. Parts of this area belong to the Circum-Rhodope belt (southern and northeastern Evros area) and to the Serbomacedonian massif (western part of the Strymon basin)^4^.

## Results and discussion

### Hydrochemistry

Chemical data and isotopic values are reported in Supplemental Table S1. Water samples have been analyzed for the major elements, lithium, boron and silica (Table S1 of the supplemental material). On the basis of the Cl contents, they may be categorized as follows:

*Low Chloride waters:* < *200 mg/l* This group includes the thermal waters of SR (NIG, SID, AGS, ACH and IRA), ND (ERA) and LFS (FYL) basins. Their temperature ranges from 18 to 62 °C. The relatively low concentration of Cl^−^ excludes the marine participation (Fig. 1).

**Figure 2:**
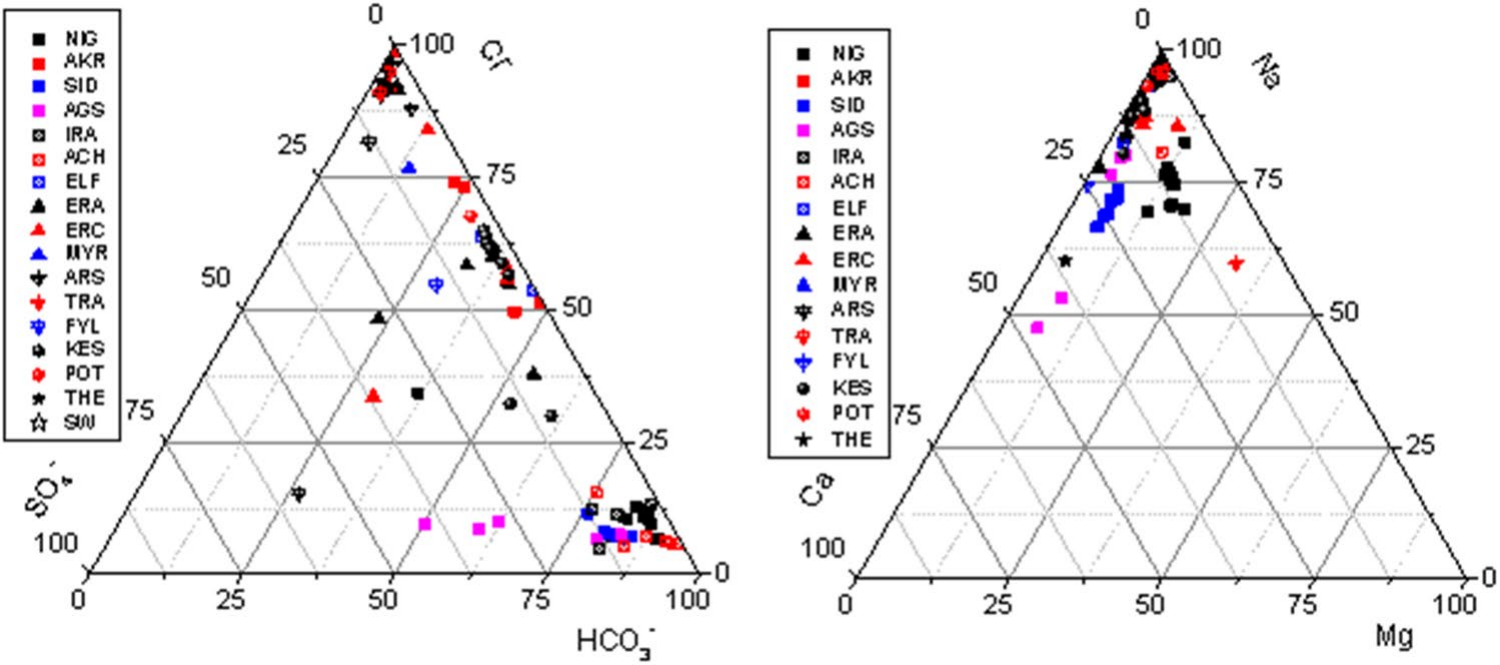
Ternary plots of Cl–HCO_3_–SO_4_ and Na–Ca–Mg for all studied areas.

*High Chloride waters:* > *200 mg/l* This group includes the thermal waters of SR (AKR and ELF), XK (KES, POT), LFS (ARS, TRA, FYL), ND (ERA, MYR, ERC) basins. Their temperature ranges from 51 to 92 °C. The relative ratios between Cl–HCO_3_–SO_4_ and the main cations Na–Ca–Mg are displayed in the ternary plots (Fig. 2) for all water samples (thermal, cold and seawater). In general, the two ternary plots show that the thermal waters have Na–Cl composition and are situated in the Cl–Na corner while the fresh waters are plotted close to the HCO_3_ corner.

Based on Cl^−^ contents it appears that the marine participation for the analyzed samples varies considerably. In order to identify the origin of chloride in this geothermal system, Cl^−^ and Br^−^^1,2,11,13–15^. The Cl/Br ratio of some high chloride samples, plotted on the seawater dissolution line, displays values ranging between 259 and 303, which are quite close to that of seawater 320. This correlation, Cl^−^ versus Br^−^, was used in order to determine the marine contribution in samples, assuming that this contribution originates from a marine component. The marine contribution calculated from O–H–Cl and Br in the deep geothermal systems varies considerably between the samples, showing that the boron concentration of these waters originates from both marine and water interaction in high temperature environments (Table S3 of the Supplemental material).

**Figure 3:**
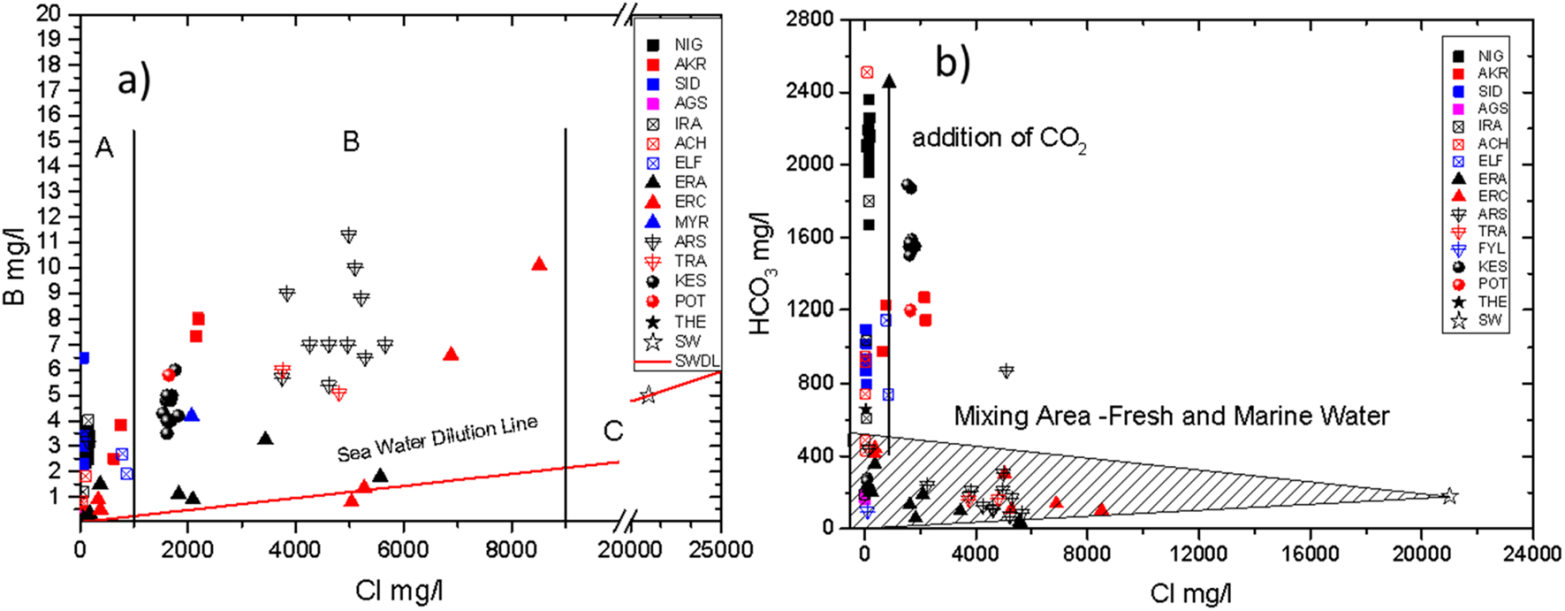
(**a**) Cl^−^ versus B for the studied areas. Area A (mixing between cold water and deep thermal water of SR), area B (mixing between cold water and deep thermal water of XK, LFS,) and line C (seawater dilution line-SWDL). (**b**) HCO_3_ versus Cl^−^ of the thermal waters for the studied areas. The shaded part of the graph indicates the mixing between fresh water of the area and marine water.

In the diagram Cl^−^ versus B (Fig. 3a), the correlations between B and Cl^−^, suggest a different origin for both elements. The ideal mixing line between seawater and fresh water (seawater dilution line, SWDL) suggests that Cl^−^ and B derive from seawater, as both Cl^−^ and Br^−^ contents showed, which is more or less diluted by fresh water and subsequently the supply of these ions by rock leaching is negligible. The majority of the other analyzed thermal waters show very variable concentrations of B (B = 0.05–11 mg/l), which is probably related to the high temperature leading to enhanced water–rock interaction and leaching of boron. Two distinct groups are observed: the thermal waters of the first group, area A, show boron concentrations between 0.05 and 7 mg/l. They exhibit high temperature (T = 30–62 °C) and Cl^−^ concentration less than 250 mg/l. The samples of this group are come from the SR basin [Nigrita (NIG), Sidirokastro (SID) and Iraklia (IRA)]. Moreover, the B/Cl and Li/Cl ratios (and Br/Cl, Cation/Cl) of most of these waters are different from those of the seawater (the B/Cl ratio of sea water sample is 2 × 10^−4^) suggesting a non-marine origin of the B and Li.

The thermal waters of the second group show similarly very variable concentrations of B (B = 1–11 mg/l), which is related to the very high temperature (T = 50–92 °C). The high B/Cl ratio, (area B, Fig. 3), for these thermal waters, SR basin [Akropotamos (AKR)], ND basin [Eratino (ERC)], XK basin [Nea Kessani (KES) and LFS basin [Traianoupolis (TRA), Aristino (ARS)] show a variability, about 3 times more than that of seawater, indicating that the excess of B can probably be of marine sedimentary origin. Apart from boron, these waters (second group plus the AKR thermal water) are also rich in Li^+^ especially the thermal waters of Nea Kessani (KES), Aristino (ARS) (the Li/Cl of ARS is 163 times greater than that of seawater) and Eratino (ERC), suggesting that water–rock interaction adds lithium and boron contents to this these thermal waters.

The condensation of CO_2_ geothermal gas enhances the influence of thermal water and thus the water–rock interaction process (the HCO_3_/Cl ratios of most of the thermal waters analyzed are higher than those of the seawater, see Fig. 3b). The observed HCO_3_^−^ in the thermal spring waters of SR (especially in Nigrita) and XK (Nea Kessani) basins is high compared to the respective fresh waters (Fig. 3b). This suggests that these thermal waters are enriched in HCO_3_^−^ (Fig. 5), probably through the absorption of CO_2_ bearing gases or by the condensation of CO_2_ of geothermal steam. In Fig. 3b we observe a gradual increase of Cl^−^ contents of the other samples. This indicates the participation of the Cl^−^ component in the deep geothermal system. For most of the thermal waters the Mg/Cl ratios are lower compared to the marine ones, a fact that indicates the interaction of the waters housed in deep volcanic formations (under high temperatures and CO_2_ pressures). In such environment, the Mg^2+^, is incorporated into secondary alteration minerals by ion exchange reaction^16^ stripping the solution of its Mg^2+^ contents. The cation–Cl ratio and the gradual increase of Cl^−^ content indicate that these waters are mixed with a deeper geothermal fluid that probably contains water of marine origin. Furthermore, the Li/Cl and B/Cl ratios are much higher than those of sea water which indicates major secondary processes during the water circulation in deep hot reservoirs. In addition, the K/Cl, Na/Cl and Ca/Cl, ratios of most thermal waters are higher compared to those of seawater, thus confirming that their salinity is controlled by water–rock interactions.

### Origin of lithium and boron

The Cl^−^, B and Li^+^ contents in fresh and geothermal waters, areas A and B (Fig. 3a, Cl vs B), as mentioned before, can be attributed to water–rock interaction (mainly for B and Li^+^) and to marine participation into the geothermal field (mainly for Cl^−^). The water of meteoric origin has a Cl/B ratio equivalent to that of seawater because these elements are originating from seawater spray and aerosols. When this water absorbs heat through deep convection, B is dissolved stoichiometrically from the surrounding rocks. The B should be removed from sediments and transferred easily to the submerged fluids; however the transfer of Li from rock requires intense water–rock interaction at high temperatures^17^. Li/B ratio of the thermal water shows wide variability from 0.09 to 1.0, which probably reflects the effects of secondary processes such as leaching of Li from terrigenous materials, in high environment temperature.

**Figure 4:**
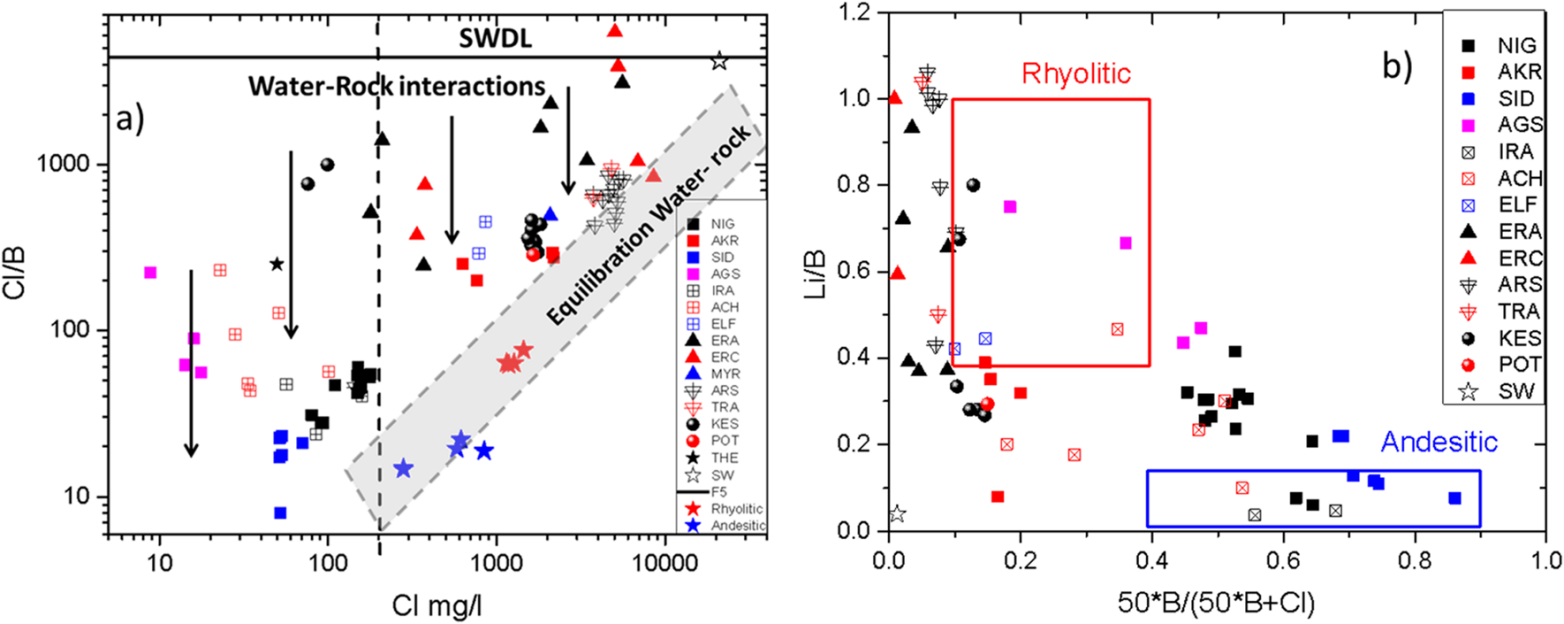
(**a**) Cl versus Cl/B of the thermal waters for the studied areas. The arrows indicate water–rock interactions. Equilibration water–rock line is depicted (shaded area-the edge points represent the interaction of thermal water with the aquifer matrix). (**b**) Li/B versus 50*B/(50*B + Cl) of the thermal waters. The rectangular shapes represent the ratios of Andesitic and Rhyolitic rocks.

As a result, the Cl/B and Cl/Li ratios of the water are getting lower than those of seawater because rocks are rich in B and Li. B and secondarily Li^+^ are present in significant amounts in igneous rocks, with their average crustal abundances close to 10–45 ppm and 6–17 ppm, respectively^17–19^. During the evolution of rock dissolution, the aqueous Cl/B and Cl/Li ratios in thermal waters are gradually approaching the rock ones, due to rock weathering. Hence, water–rock interaction adds a significant amount of B and Li to geothermal waters, but only a very limited amount of Cl^−^, since rocks do not contain important quantities of Cl^−^ (except from evaporites). Consequently, all thermal waters exhibit Cl/B and Cl/Li ratios lower than seawater (SW). However, the thermal waters of Eratino (ND) and Aristino (LFS) exhibit Cl/B ratio lower than seawater but higher than the rest of the analyzed thermal waters. The B excess with respect to Cl, in these thermal waters, is due to the contribution of seawater (Fig. 4a Cl vs Cl/B). When the contribution of seawater is important, geothermal water similar to those encountered in the Eratino and Aristino fields (80–99 °C with Cl > 200 mg/l) is susceptible to be generated.

In Fig. 4a three members are distinguished: fresh water (sea water dilution line), thermal water of meteoric origin (Cl < 200 mg/l) and thermal water by mixing (either with marine or meteoric water). Thus, the potential source of B in the study area is related mainly to the recharge of meteoric water and to the interaction of water with the aquifer matrix. Marine contribution represents only minor localized contributions of B. The same observations are also valid for Li contributions. Furthermore, the Li/B ratio of thermal waters shows wide variability (0.001–1), typical of waters encountered in geothermal systems. The high Li/B ratio of Eratino, Aristino and Nea Kessani fields also exhibits wide variability from 0.4–1 to 0.3–0.67 respectively, typical of waters discharged from volcanic systems^19^. The Li/B ratio of Nigrita (0.06–0.4), Akropotamos (0.08–0.4) and Loutra Elefteron (0.4) thermal waters are also in the range of volcanic systems^1,11,19^. The maximum Li/B ratio is observed in Aristino and Nea Kessani geothermal fields. The intensity of the water–rock exchange depends, among other parameters, on the relative proportions of water and rocks, on the surface area and the duration of the contact. This variation observed from Nigrita to Nea Kessani and Aristino fields is consistent with the tensional tectonic regime and the water age that both increase from west to east. Probably the supply of B^+^ and Li^+^ ions by rock leaching removal process is compatible with geothermal fluids alteration occurring in the crustal zone during emerging and resurfacing of the waters. In order to evaluate the nature of the hosting rocks all data points and the ratios of Andesitic and Rhyolitic rocks^19^ are shown in Fig. 4b.

In Fig. 4b, the distribution of all data points supports the above classification into two distinct groups, based on chloride contents: a high-50*B/(50*B + Cl) (~ 1), Cl-rich thermal water, as represented by Aristino and Nea Kessani, and a low-50*B/(50*B + Cl) (< 0.4), as represented by Nigrita. The other sites occupy intermediate positions. Possible source of both B and Li may be the geothermal systems hosting rocks of intermediate to acid composition (andesites to rhyolites and their plutonic equivalents), as represented by the Nigrita and Aristino–Nea Kessani fields, respectively.

### Isotope analysis

#### Origin of CO_2_ gases

To evaluate the CO_2_ origin, the δ^13^C_DIC_ of thermal waters was analyzed. The δ^13^C_DIC_ reflects the different origin of CO_2_ gas and DIC that can be related to the atmosphere, geological processes, lithology and other exogenous sources.

In shallow carbonate aquifers, the δ^13^C_DIC_ in groundwater ranges between − 10 and − 22‰ (Please refer to Figure S2 of the Supplemental material), indicating mixing processes between soil—CO_2_ (δ^13^C value around − 22‰, C3-temperate climate) and CO_2_ produced by carbonate water–rock interaction (δ^13^C δ^13^C =  − 2 to 1‰)^20,21^. In silicate aquifers the chemical reactions that took place during the deep circulation of water in this hydro-lithological system produce an insignificant shift in δ^13^C_DIC_^22^) producing water whit δ^13^C_DIC_ that ranges between − 22 and − 8‰.

**Figure 5:**
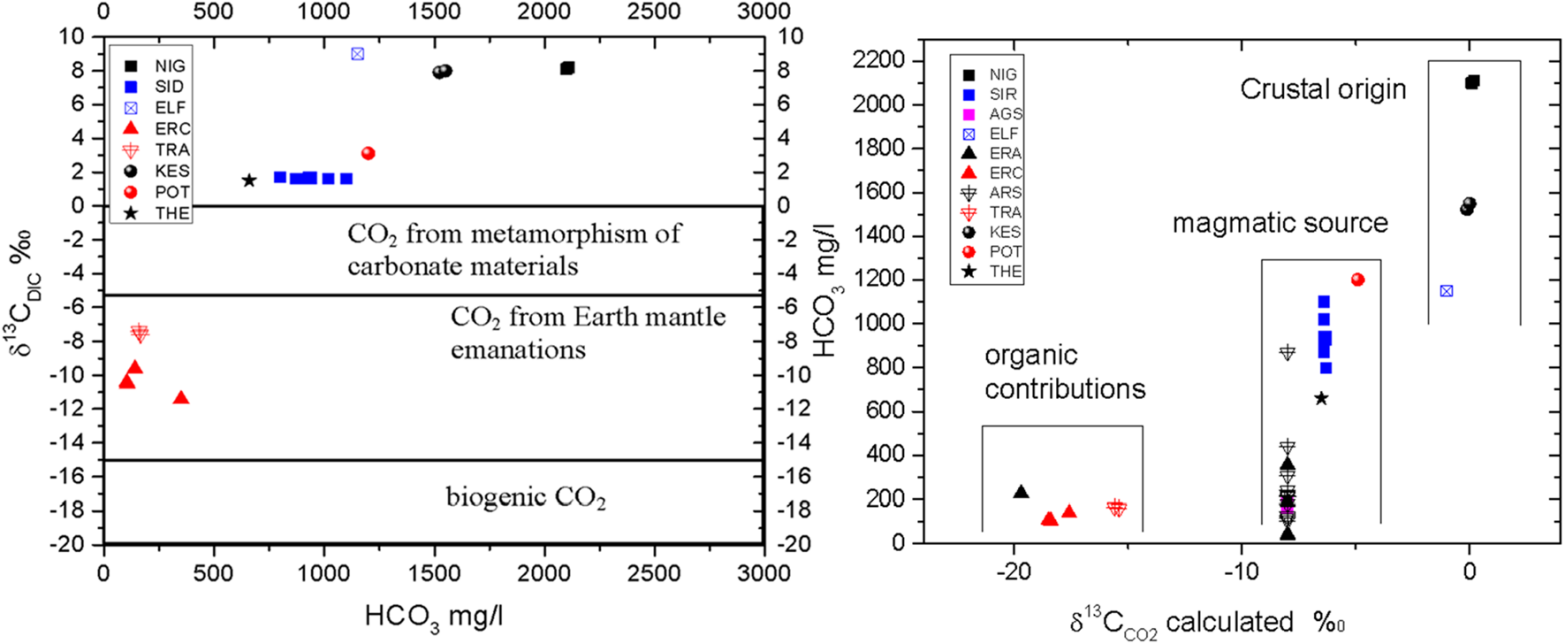
(**a**) δ^13^C_DIC_ versus HCO_3_ of the thermal waters for all studied areas. (**b**) δ^13^C_CO2 calc_ versus HCO_3_ of the thermal waters for the studied areas.

For groundwater that circulates in deep aquifers with long residence time, the carbon chemistry results from mixing procedures between: (1) deep CO_2_ with ^13^C value between − 8 and 3‰; (2) CO_2_-gas from atmosphere (3) biogenic CO_2_ with ^13^C value between − 10 and − 22‰ and (4) mineral C with ^13^C content at + 0‰ (Fig. 4a). In such aquifers, the carbon mass balance is related to the rate of water–rock interactions under high temperature and to fractionation factors. In nature, this is represented with δ^13^C_DIC_ contents around − 8‰^22^. The geochemical processes, which can produce large amounts of deep origin CO_2_, are associated with magmatic-volcanic fluids, thermal or metamorphic decarbonation of carbonate rocks (limestones) and diagenesis of organic sediments. The δ^13^C_CO2_ values of gas originating from earth mantle emanations range between − 4 and − 8‰^23,24^. The marine limestone, with ^13^C value between − 2 and 1‰, metamorphism produces δ^13^C_CO2_ about 0‰ ± 3‰^20,23^, while the gas originating from the thermal decomposition of crustal carbonate rocks has values between − 4 and 0‰^25^. The carbon originating from diagenesis of organic sediments shows δ^13^C_CO2_ around − 30‰^23^ (Fig. S2 of the Supplemental material & Fig. 5a).

The δ^13^C_DIC_ values of the collected thermal waters ranges from 9 to − 12‰ (Fig. 5a). In order to assess the system in which the C evolves and to better discuss the carbon origin in conjunction with the relationship of magmatic versus crustal contribution, a calculation of δ^13^C of the gaseous phase in equilibrium (^13^C_gas eq_) with measured DIC has been carried out using adequate equation and the fractionation factors^26^. The calculated δ^13^C contents of the CO_2_ in equilibrium with the DIC vary between − 19.7 and 0‰. Daskalopoulou et al. (2016)^27^ measured the δ^13^C of CO_2_ contents from the Rodope massif with values very similar to those computed.

Considering that the isotopic value of gas of deep origin originating from earth mantle emanations (δ^13^C values around − 6‰) and the gas originating from “crustal”, thermal or metamorphic decarbonation of limestones, (δ^13^C_CO2_ values close to 0‰ ± 3‰) the thermal waters from Sidirokastro (− 6.3‰), Potamia (− 4.9‰), Aristino (− 8.0‰), Erasmeio (− 8.0‰) Agistro (− 8.0‰) and Thermes (− 6.5‰) produce results of δ^13^C typical of magmatic source. In the case of Nigrita (0.2‰), Loutra Elefteron (− 1.0‰) and Nea Kessani (0‰) fields, the carbon source could be associated to “crustal” component, thermal or metamorphic decarbonation of limestone, (Fig. 5b). Conversely, the carbon source of the samples from Erasmio12 (− 19.7‰), Eratino (from − 19.4 to − 17.6 ‰), and Traianoupolis (− 15.6‰) indicate that carbon reflects organic contribution.

#### Stable isotopes of water

**Figure 6:**
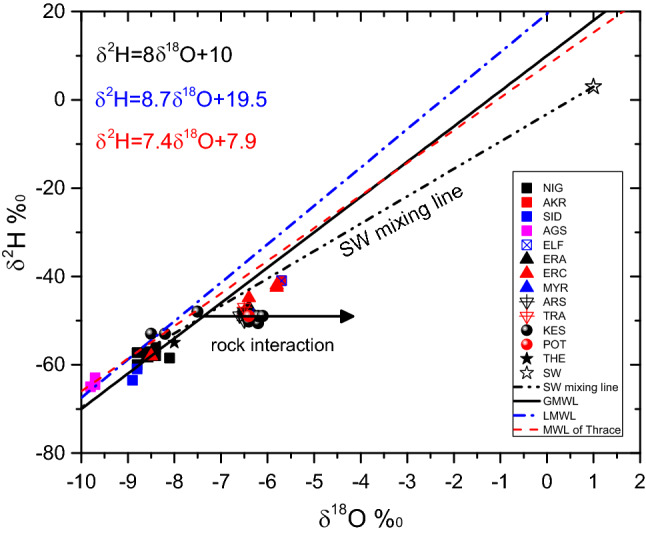
δ^18^O versus δ^2^H of the thermal waters for all studied areas. The black solid line is the Global Meteoric Water Line, The blue dash-dot line is the Local Meteoric Water Line (δ^2^H = (8.7 ± 0.44)*δ^18^Ο + 19.5 ± 3.1) and the red dash line is the Meteoric Water Line of Thrace (δ^2^H = (7.39 ± 0.37)*δ^18^Ο + 7.9 ± 3.0).

The stable isotopic composition (δ^2^H versus δ^18^O) of all waters is shown in Fig. 6^28,29^. Some waters samples from Nigrita and Sidirokastro geothermal fields are localized in the group of fresh waters along the Local Meteoric Water Line for Greece (LMWL)^28^ (Fig. 6), indicating a meteoric origin without the participation of seawater, as it is concluded by the geochemical analysis. Contrary, the points representative of the SR basin (Akropotamos) and respectively those of ND basin (Eratino, ERC-4 and 8) are slightly shifted to the right of the LMWL and fall to the line that represents an ideal mixing between seawater and local fresh water. This is originated through dilution and addition of local groundwater to a deep, hot geothermal liquid of marine origin. The majority of these samples have been plotted onto the mixing line confirming the ratios of sea water participation as calculated by Cl contents (Table S2 of the Supplemental material). However, the mixing between the SR (Akropotamos) and ND (Eratino) basins thermal fluids and seawater is favored by the location of the waters discharge on the shoreline. The occurrence of this process is also confirmed by ^18^O and Cl contents (Table S2 of the Supplemental material).

The points representing the thermal water from Nea Kessani fall to the right of a ‘mixing’ point resulting from mixing between fresh and marine water. The deuterium content of these thermal waters is − 50 ± 1‰, as expected for waters originating from the mixing of cold [− 53‰ (samples KES-6; KES-7), at about 95%]) and sea water (4‰, at about 5%). Nevertheless, their δ^18^O value ranges from − 6.2 to − 6.4‰, which is significantly different from what is expected [-8.2‰ (samples KES-6; KES-7)] from the above mixture percentages. Also, the δ^18^O content of the Nea Kessani thermal water is below the LMWL indicating significant δ^18^O enrichment with respect to “mixing” water and suggesting an isotopic exchange with the rocks of the geothermal reservoir. In order to approach the isotopic equilibrium state, between water and rocks, corresponding to the temperature of the geothermal reservoir, we consider that the liquid phase is enriched in oxygen isotope. This process affects only the oxygen, because the Deuterium content of rocks is too low to significantly modify the isotopic composition of thermal fluids. The horizontal line that deviates from the mixing point and plotted in the mixing line between fresh and marine water, to higher δ^18^O values (− 6.2% to − 6.4‰), represents an ‘oxygen shift’ caused by the previously mentioned water– rock interaction process. This positive oxygen shift, at least of 1.5‰, seems to occur in areas of high thermal potential of the geothermal reservoir. A similar oxygen-sift is compatible with water–rock interaction at high temperatures (200 °C). The existence of high temperature in the geothermal reservoir is also supported by the high Li (and B) content, since the Li transfer from reservoir rocks requires intense water–rock interaction at high temperatures. This Li/B variability probably reflects intermediate maturation steps towards the composition of average reservoir rocks. All the used geothermometers show such high temperatures at Nea Kessani area.

This isotopic enrichment is also observed in the thermal waters of LFS and ND (ERΑ-9), although these waters are the result of a mixture of sea and meteoric water (Table S3 of the Supplemental material). So their representing points would be expected to be on the marine dilution water line and that they would not be shifted to the right (in respect of the dilution water line). Their shift to the right, which isn’t so pronounced in relation to Nea Κessani, is attributed also to water–rock interaction at relatively high temperatures. Their Li and B contents are also in favor of this case.

#### Stable isotope of sulfate

The ^34^S and ^18^O contents of the analyzed samples (Table S2 of the Supplemental material) are compared to those of marine sulfate (δ^34^S = 20.1‰ CD and δ^18^O = 9.3‰ SMOW)^30^. In contrast, the isotopic ratios of sulfates in rain-water are dependent on their origin (the isotopic ratios of rain- water show a variability between 5 and 17‰ for δ^18^Ο^31^ and − 2.5 to 19.5‰ for δ^34^S^32^).

The δ^34^S and δ^18^O values of all thermal waters are very different to those of marine sulfate. The water samples from Nigrita, Sidirokastro, Agistro, Thermes, Potamia and Nea Kessani are of meteoric origin, without any significant participation of seawater, while the waters from LFS basin (Aristino and Traianoupolis) show a marine participation lower than 25%. Whatever the origin of water is, the δ^18^O values (1.5 to 7.5‰) of the thermal waters are very different from those of marine sulfate (δ^18^O = 9.3‰).

**Figure 7:**
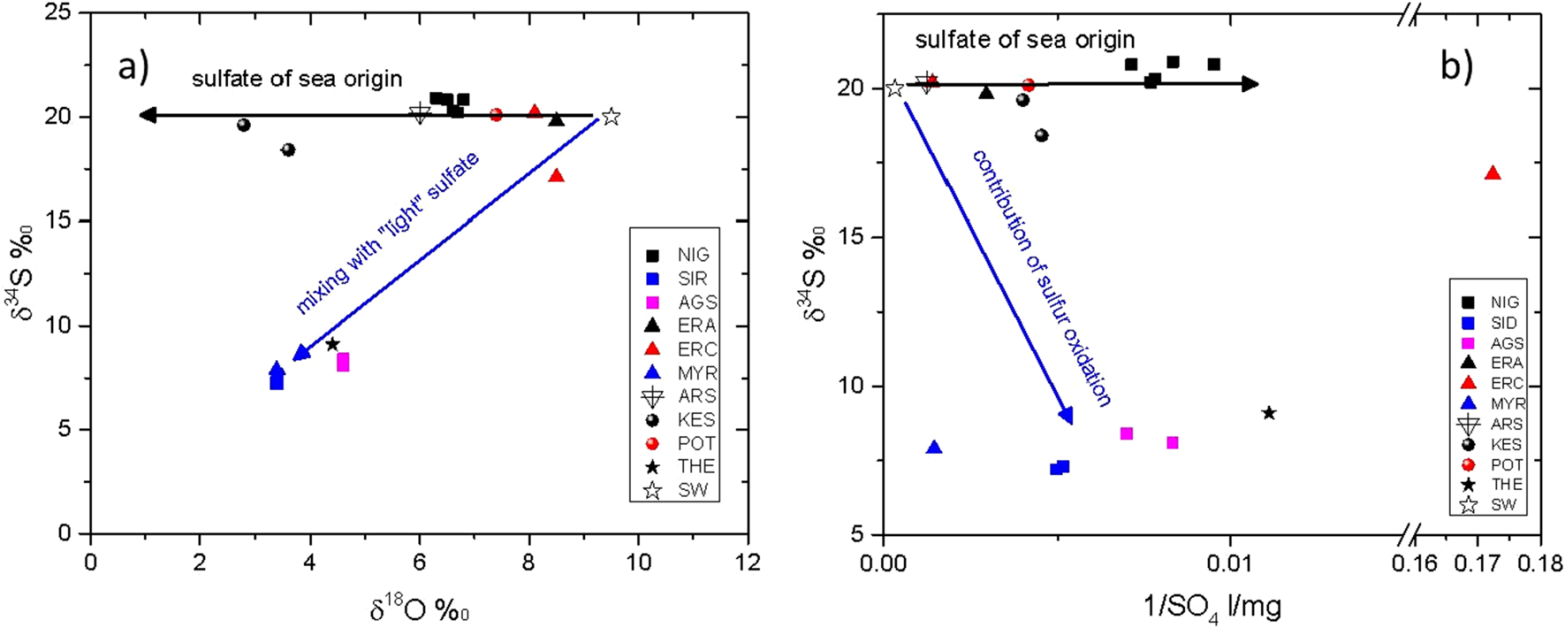
(**a**) δ^18^O versus δ^34^S, values of the thermal waters for all studied areas. (**b**) 1/SO_4_ versus δ^34^S of the thermal waters for all studied areas.

The δ^34^S, δ^18^O and SO_4_ values of thermal waters from Sidirokastro and Agistro (Fig. 7a, b) are too low compared to the SO_4_^2−^ corresponding values of marine water. This fact indicates that the SO_4_^2−^ in the water of these springs results from the mixing of aerosol (marine sulfate) with “light” sulfate that is transported by the meteoric component. This “light” sulfate probably originates from dissolution of sulfur of deep rocks (average value δ^34^S = 0‰ to − 12‰)^31^ or from mixing of sulfates resulting from sulfur oxidation. The contribution of sulfur oxidation is confirmed by the increase of 1/SO_2_^4−^ ratio (0.25 versus 0.0003 of seawater), as indicated in Fig. 7b.

The sample (ERC-4) has the lowest SO_4_^2−^ content measured in ND basins and its δ^34^S is lower than those of seawater. This low content in SO_4_^2−^, suggest that its SO_4_^2−^ has been almost totally reduced. These values are so different from marine sulfates that any possibility for marine water participation is excluded. The δ^18^O of SO_4_^2−^ is influenced by the intervention of the phenomenon of reduction as well as the interaction of the ^18^O of water with the ^18^O of aqueous sulfate. In fact, not only the δ^18^O value of the SO_4_^2−^ in this sample but also the reduced values of δ^18^O in the water of the sample ERC-4 (8.5‰) may also be attributed to isotopic exchange between the oxygen of the water molecules and that of the sulfate ions.

The δ^34^S value of the rest of the thermal water samples shows that their ^34^S content (δ^34^S = 18.4–20.9‰) and respectively their Cl/SO_4_ ratios are similar to those of the SO_2_^4−^ in seawater, indicating the seawater origin of these anions. The reduced δ^18^O value of the SO_2_^4−^ in the samples of thermal waters (2.5–8.5‰), comparing to the corresponding SO_2_^4−^ value of seawater origin (9.3 ± 0.2‰) may be attributed to isotopic exchange, in high temperature, between the oxygen contained in the water molecules and in the sulfate ions [δ^18^O–(H_2_O–SO_4_)]. This oxygen variation seems to reflect an equilibrium process more (Nea Kessani–Aristino samples) or less (Nigrita, Erasmio samples) completed.

### Geothermometry

For evaluating the reservoir temperatures, the following geothermometers: SiO_2_^33^, Li–Mg^34^, Na–K^35^, Na–Li^36^ and Na–K–Ca^37^ were applied. In general, the temperatures obtained with the Na–K and Na–K–Ca geothermometers (Table 1) are higher than those obtained with the other geothermometers. Generally, Watek-F thermodynamic model indicates that most of the studied waters are oversaturated (> 0.8) as far as silica is concerned, and therefore the temperatures obtained represent the lowest range attributable to the deep hydrothermal waters. Na–Li and Li–Mg geothermometers also show temperatures close to those obtained by applying the silica geothermometers.

**Table 1:** Reservoir temperatures, as calculated from geothermometers.

Basin	Location	SiO_2_	Na/K	Na/K/Ca	Na/Li	Mg/Li	^18^OSO_4_–H_2_O
*SR*	*Nigrita*	100–140	80–270	140–250	90–140	60–100	120–130
	*Akropotamos*	120	75–195	140–225	20–105	70–105	
	*Sidirokastro*	80–120	90–265	130–240	80–120	70–80	150
	*Agistro*	45–110	130–170	70–160	80–150	65–80	130
	*Iraklia*	60–110	90–150	150–200	30	60–75	
	*Ivira–Achinos*	55–75	45–80	90–135	20–45	55–90	
	*Loutra Elefteron*	100	170–190	195–210	85–110	90	
*ND*	*Erasmio*	60–120	60–170	90–195	45–110	75–120	125
	*Eratino*	70–90	60–135	120–180	20	75	125–200
	*Myrodato*		170	200			190
*LFS*	*Aristino*	70–130	60–170	95–200	75–190	55–120	150
	*Traianoupoli*	95	120–135	170	75–125	55–105	190
	*Fylakto*		75	120	190	120	
*XK*	*Nea Kessani*	80–115	80–225	130–240	70–110	80–105	180–200
	*Potamia*		200	220	90	95	140
*RM*	*Thermes*		230	200			150

The application of Na–K and Na– K–Ca geothermometers for the samples from the SR and ΧΚ basins indicates temperatures ranging between 45–275 °C and 60–245 °C respectively. These temperatures are overestimated and this is probably due to the precipitation of carbonate minerals during the up flow of water to the surface under high temperatures. In fact, all the studied waters are saturated with calcite. The latter is confirmed by the presence of extended travertine deposits in the area. The application of Na–Li (40–140 °C) and Li–Mg (60–110 °C) geothermometers give temperatures close to those calculated by the silica geothermometers (50–140 °C) that are the minimum accepted for these fields.

The resulting temperature for ND and LFS basins is different among the various geothermometers applied, which is more evident from sample to sample. The contribution of marine solutions to the deep thermal fluids is one of the main causes of the disturbance of chemical and isotopic geothermometers. Additionally, the SiO_2_ geothermometer (70–130 °C) that is not affected by the marine contribution does not agree with the maximum temperature of 170 °C and 200 °C estimated by the Na–K and Na–K–Ca geothermometers, respectively. Furthermore, the Na–Li (60–170 °C) and Li–Mg (70–130 °C) geothermometers also exhibit values much lower than those taken by the Na–K–Ca but similar to those of the SiO_2_ and Na–K geothermometers. In all, the temperature obtained by the use of SiO_2_ geothermometer is considered to be the lowest one, attributable to the deep hydrothermal waters (ND and LFS basins).

Conclusively, the use of these chemical geothermometers is very ambiguous, except for the SiO_2_ geothermometer, since they are not representative of water–rock equilibrium. The sulfate–water isotopic geothermometer^38,39^, which is based on the equilibrium exchange of oxygen isotopes between aqueous SO_4_^2−^ and H_2_O, was used for the comparison with the chemical geothermometers. The ^18^O content of aqueous sulfate depends on the contribution of oxygen, which participates in the SO_4_^2−^ ion, the oxidation fractionation factors, and the equilibrium between ^18^O of SO_4_^2−^ and ^18^O of water.

The calculated temperatures for the wider sampling area, were variable and dependent on the sampling location [NIG (120–130 °C) < KES (180–200 °C); SR basin] and from spring to spring (ex. LFS springs = 150 °C to 200 °C).

The δ^18^O values of thermal waters from the LFS (Aristino, Traianoupolis), the XK (KES, POT) and the SR basin (NIG-4, NIG-6 to 8, NIG-11) are much diminished in relation to that of seawater. The decrease of δ^18^O value of the SO_4_^2−^ in the water of these springs (from 2.8 to 7.4‰) in comparison with the SO_4_^2−^ of sea water origin (9.3 ± 0.2‰) could be attributed to the isotopic equilibration between δ^18^O_(H2O)_ and δ^18^O_(SO4_^2−^_)_. This oxygen variation seems to reflect an equilibrium process more (Aristino and Nigrita samples) or less (Potamia) completed. In particular, if the δ^18^O content of aqueous sulfate of these samples is only controlled by equilibration with water, and if isotopic equilibrium is reached, the δ^18^O (SO_2_^4−^–H_2_O) temperature will better express the deep geothermal temperature. In fact, the calculated temperatures are 120 °C and 200 °C respectively. The advanced equilibration process of KES-2 and KES-5 (δ^18^O = 2.8–3.6‰) is followed by TRA-2 (δ^18^O = 3.1‰) and ARS-4 (δ^18^O = 6.0‰), NIG-4,6-8,11 (δ^18^O = 6.3–6.8‰) and POT (δ^18^O = 7.4‰). The equilibration between δ^18^O (SO_4_–H_2_O) in NIG-4 (6.8‰) and POT (δ^18^O = 7.4‰) water isn’t so pronounced in relation to the other deep geothermal fields and this appears in the calculated temperature. The temperature obtained for NIG-4 and POT is significantly reduced (120–140 °C) in relation to LFS and XK geothermal fields. The δ^18^O content of the SID-6, 7, AGS-1,2, MYR and THE water samples was the result of mixing between sulfate of marine origin and sulfate resulted from sulfur oxidation and consequently the temperatures obtained by the use of this isotopic geothermometer, ranging between 130 and 190 °C, are questionable. The calculated temperatures, by the isotopic geothermometer, for the Eratino (ERC-4) are questionable because partial reduction of aqueous sulfate has been detected and as a result the suggested temperatures are underestimates. In fact, since it is not possible to quantify the reduction processes, the estimated temperature is considered to be the lowest acceptable for the deep reservoir. If isotopic equilibrium was achieved, then the temperature of deep thermal water would be greater than 200 °C.

Thus, the isotopic geothermometer suggests a probable existence of a deep geothermal field of low to middle enthalpy (120–130 °C) for SR (NIG samples) and ND (ERA samples) basins and of middle to high enthalpy (150–200 °C) for Eratino (ERC-4), LFS (ARS, TRA samples) and XK (KES samples) basins.

## Conclusions

The enrichment in δ^18^O, δ^2^H and the Cl^−^ content, are significant parameters to assess the participation of marine water in geothermal systems. These isotope-chloride relations suggest the contribution of marine water into the deep geothermal reservoirs as observed at ND (ERC, ERA), LFS (ARS and TRA) and SR (only AKR samples) basins. In contrast, in the XK basin (KES), groundwater show ^18^O shifted through isotope exchange with rocks at high temperature. Regardless the marine participation, there is an increase in lithium concentration from the west (SR Basin) to the east (LFS basin) showing an intense water–rock interaction at high temperature. In addition, the equilibration between the oxygen of the water molecules and the one of the sulfate ions [δ^18^O–(H_2_O–SO_4_)] may be also referred to isotopic exchange, at high temperature.

**Figure 8:**
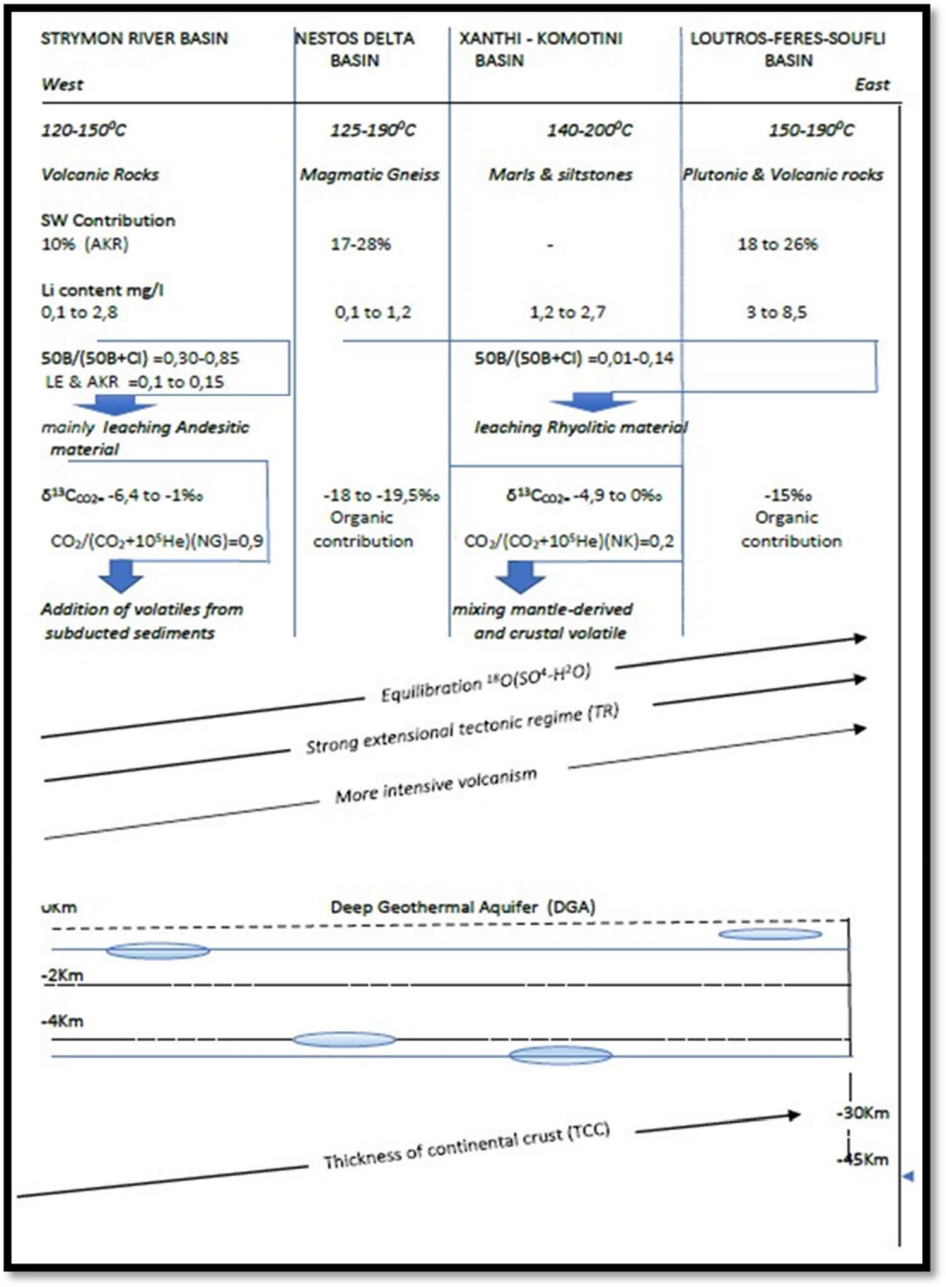
Schematic diagram summarizing the relevant characteristics of geothermal fields in North and Northeastern Greece. *See Shimizu et al. (2005)^44^.

Figure 8 is a schematic diagram summarizing the relevant characteristics of geothermal fields in North and Northeastern Greece, showing the causes that could elucidate the variation of deep temperatures and geochemical processes. In Greece the recent and intensive volcanism has contributed to the existence of many thermal manifestations associated with the back-arc regions of SR, ND and LFS basins^40–42^. These back-arc geothermal fields are complied with extensional tectonics resulting in the crust thinning and the formation of faults, which both favors the quick rise of deep thermal fluids^40^. The thickness of the continental crust under *Loutros–Feres–Soufli basin* is less than 35 km, while it is 40–45 km beneath Strymon River basin^43^.

This geological and geodynamic setting of each geo-lithological basin left its trace in the isotopic and chemical compositions of thermal waters, in the heat flow values and in the CO_2_ concentrations: from the west to the east part of the studied areas both B and Li are derived through leaching of rocks with intermediate to acid composition (andesitic to rhyolitic material and their plutonic equivalents) respectively. These hosting rocks of the geothermal systems are responsible for the B and Li contents analyzed in the deep thermal waters, and their heat regime. Additionally, the δ^13^C isotopic ratio of gas shows a mixing contribution of crustal constituents and mantle components and for the West part, an addition of volatiles^44^ from subducted sediments cannot be excluded.

The isotopic geothermometers reflect also, for the same direction, an equilibrium process more (*LFS and XK basin*) or less (SR basin) pronounced and discriminate the geothermal field from low to middle [SR and ND (ERA) basins, located at 1200 m and 500 m respectively] and middle to high enthalpy [ND (ERC), LFS and XK basins, located at 4500 m, 700 m and 4500 m respectively].

The differences encountered in the isotopic-chemical compositions and the thermodynamic characteristics of the sampled fluids, from west to east, are probably due to the tectonic regime, to the depth of the deep reservoirs, to the age of magmatic activity and in general to the geomorphological situation of each basin.

## Sampling and analytical methods

Thermal waters (springs and wells) and sea water were sampled in geothermal areas of North and North East Greece (Table S2 of the Supplemental material). Temperature, pH, conductivity and alkalinity were measured directly in the field.

For the chemical analysis, water samples were collected in polyethylene containers of 700 ml. Two samples of waters were taken, one acidized (HNO_3_ 1:1) for cation analysis and one not-acidized for anion analysis. Samples were analyzed at the Institute of Geosciences and Earth Resource, C.N.R., Pisa and I.G.M.E. Moreover, for the isotopic analyses of water (^18^O, ^2^H), carbonates (^13^C) and sulfates (^18^O_SO4_, ^34^S), samples were taken separately in two-glass bottles (50 ml and 1 l) at every sampling point.

The analysis of major chemical constituents was carried out according to the standard methods described in Alpha (1989). SiO_2_ contents were determined by atomic absorption. Anions were analyzed by ion chromatography. The B content was determined photometrically using the curcumin method. The samples were acidified and evaporated to dryness (at 55 °C) in the presence of curcumin. The precipitate is red in color and can be dissolved in ethyl alcohol. The red alcoholic mixture was photometrically determined at 540nm^45^. The chemical analysis took place at the Institute of Geosciences and Earth Resource, C.N.R.-Pisa. Minor element (Li) was measured using inductively coupled plasma optical emission spectrometry with a precision of better than ± 2%. Silica contents were determined by atomic absorption with a precision of ± 2%, (404A Method of Standard Methods for the Examination of Water and Wastewater, APHA-AWWAWPCF, 19th edition, 1995). The isotopic composition of oxygen (δ^18^O), hydrogen (δ^2^H), carbon (δ^13^C), and sulfate (δ^34^S), was measured at the Stable Isotope laboratory of the Institute of Nanoscience and Nanotechnology of the National Center for Scientific Research “Demokritos” (Athens, Greece) by a Thermo DELTA V plus IRMS (Isotope Ratio Mass Spectrometer) coupled with Gas Bench II and Flash Elemental Analyzer (Thermo Electron Corporation, Bremen, Germany). δ^18^O and δ^2^H were determined in water samples using the CO_2_–H_2_–water equilibration method^46,47^. The dissolved inorganic carbon in the water, for the analysis of ^13^C, was collected as BaCO_3_ precipitate. Carbon and oxygen isotope analyses of samples were carried out using the conventional phosphoric acid procedure and the continuous flow technique^48^. The dissolved SO_2_^4−^ in the water samples was precipitated as BaSO_4_ for the analysis of S isotopic compositions^49^.

The isotope composition of ^2^H, ^18^O, ^13^C and ^34^S is indicated in delta notation, versus VSMOW, VPDB and CDT standards, respectively, as:

$$\updelta = \left[ {\left( {{\text{R}}_{{{\text{sample}}}} - {\text{R}}_{{{\text{standard}}}} } \right){\text{/R}}_{{{\text{standard}}}} } \right]*1000$$

where R_sample_ and R_standard_ refer to ^2^H/^1^H or ^18^O/^16^O or ^13^C/^12^C or ^34^S/^32^S ratios of samples and standard respectively. Determination of the different isotope ratios has the following precisions: ± 1‰ for δ^2^H and δ^34^S; ± 0.2‰ for δ^18^O_s_; and ± 0.1‰ for δ^18^O of water and ^13^C.

## Supplementary Information

Supplementary Information.

## Data Availability

Data are available from the corresponding author upon request.
