# Supplementary material for: Chemical and isotopic characterization of the thermal fluids emerging along the North–Northeastern Greece
Source: Sci Rep. 2021 Aug 11;11:16291. doi: 10.1038/s41598-021-95656-6 (PMC8357817; doi:10.1038/s41598-021-95656-6)
Supplement: Supplementary file 1 — Supplementary Information. [file 41598_2021_95656_MOESM1_ESM.docx]

**Supplemental Information of the manuscript “Chemical and isotopic characterization of the thermal fluids emerging along the North-Northeastern Greece”**

**^1, 2,*^Dotsika E., ^3^Dalampakis P., ^4^Spyridonos E., ^1^Diamantopoulos G., ^1^Karalis P., ^1^Tassi M., ^2^Raco B., ^5^Arvanitis A., ^6^Kolios N., and ^7^Michelot J. L.**

^1^Stable isotope Unit, N.C.S.R. “Demokritos”, 15310, Ag. Paraskevi Attikis, Greece

^2^Institute of Geosciences and Earth Resources, C.N.R.,Via G. Moruzzi 1, 56124 Pisa, Italy

^3^[Hellenic Agricultural Organization - Demeter](https://www.researchgate.net/institution/Hellenic_Agricultural_Organization-Demeter), [Soil and Water Resources Institute - Sindos](https://www.researchgate.net/institution/Hellenic_Agricultural_Organization-Demeter/department/Soil_and_Water_Resources_Insitute-Sindos)

^4^PPC Renewables S.A, 15343 Ag. Paraskevi Attikis, Greece

^5^Hellenic Survey Of Geology And Mineral Exploration GR

^6^ Institute of Geology and Mineral Exploration (IGME), Thessaloniki, Greece.

^7^Géosciences Paris Saclay (GEOPS), Université Paris Saclay - CNRS, France

*Correspondence to: e.dotsika@inn.demokritos.gr

**Information on the studied areas**

In Northern Greece the geothermal exploration began in the 1970’s with an investigation carried out by I.G.M.E. (Institute of Geology and Mineral Exploration). During this stage, the well of Fylakto-Tychero area produces Na-Cl type geothermal waters with minimum flow rate of 200 m^3^/h and temperature of 37,0°C, while water temperatures up to 99°C and flow rates higher than 100m^3^/h have been measured during wells production tests in the Aristino-Traianoupoli area [^1^](#_ENREF_1).

The eastern and central part of the Xanthi-Komotini basin is characterized by a relatively elevated heat flow regime and the subsequent presence of geothermal aquifers with low water temperatures ranging between 30°C and 45°C at depths 400-500 m. On the contrary, on the western margin of the basin and most precisely in the area of Nea Kessani-Xanthi, the main hot reservoir is found at shallow depths (150-400 m) with water temperatures ranging from 75 to 82°C [^2^](#_ENREF_2). In Nestos Delta basin the maximum depth of the 22 exploration boreholes drilled by I.G.M.E. reaches 500m. The values of the geothermal gradient are very high (up to 25°C /100 m). The water temperatures range from 40 to 72°C. The geothermal gradient for the Strymon basin has been estimated to fluctuate from 25 to 36oC/km at depths over than 2000 m [^1^](#_ENREF_1).

**Geology**

**a. Outline of the geology of North Eastern Greece**

The wider region of northeastern Greece is extending between the Strymon River in the West and the Evros River in the East (Greek-Turkish borders). The geological evolution of North Eastern Greece is related to the Late Cretaceous closure of the Vardar Ocean when subduction related magmatism developed in the Eastern European margin causing the consumption of different portions of Tethys Ocean (e.g., Stampfli and Borel 2004)[^3^](#_ENREF_3). It belongs mainly to the crystalline mass of the Rhodope Massif. Parts of this area belong to the Circum-Rhodope belt (southern and northeastern Evros area) and to the Serbomacedonian massif (western part of the Strymon basin)[^2^](#_ENREF_2).

*The Hellenic Rhodope Massif (HRM)* is a polymetamorphic terrain representing an intermediate continental zone accreted to the southern European margin during the Maastrichtian-Paleogene closure of the Vardar Ocean together with the Serbo-Macedonian Massif [^4^](#_ENREF_4). The onset of the Rhodope extension has been placed, by several authors, in the Early-Late Eocene– Oligocene [^5-11^](#_ENREF_5) and continued with extensional faulting and magmatism in the Oligocene-Early Miocene [^12^](#_ENREF_12). The Rhodope massif consists of gneiss, marbles and mica schists. Tertiary granitoids intruded the Rhodope massif from Eocene to Miocene [^2^](#_ENREF_2).

*The Circum-Rhodope Belt (CRB)* comprises a Late Paleozoic and Mesozoic marginal volcano-sedimentary narrow belt, bordering both the HRM to the southeast and the CRB to the west [^12^](#_ENREF_12). It consists of Mesozoic low-to-medium-grade metamorphic formations (phyllites, green-schists and post-sediments of an old flysch with intercalated eruptive and effusive volcanic bodies extending from the central and southern Evros region to the island of Samothrace.

According to Marchev et al. (2004) [^7^](#_ENREF_7) in the Rhodope Massif the onset of the extensional phase started at the Middle Eocene with fast exhumation and formation of core complexes, and continued with steep extensional faulting and magmatism in the Late Eocene-Oligocene [^4^](#_ENREF_4). During this phase and consequently to the segmentation of the Rhodope orogen, the Tertiary evolution of the HRM and the CRB is characterized by the presence, at their margins and on them, of fault-controlled (depression) sedimentary basins, formed under tensional tectonics. The formation of these basins, with the associated magmatic bodies (plutonic and volcanic rocks), started in the Middle Eocene (Lutecian) and lasted up to the Pliocene [^12^](#_ENREF_12).

In the case of the Evros River and Xanthi-Komotini Tertiary basins, they are mostly filled by Low-middle Eocene-Upper Oligocene molassic sediments. Neogene and Quaternary sediments have been deposited later in all the basins of Northern Greece.

The Tertiary sedimentary basins of Northern Greece have been thermally activated during Eocene consequently to the collision of Eurasian and African lithospheric plates. The crustal thinning in the back-arc basin as a result of extensional tectonics, have been followed by an up rise of heat flow and shift of isotherms to the surface [^13^](#_ENREF_13). The latter was intensified by the synchronous occurrence of an intense calc alkaline type volcanic explosive and effusive activity. As a result of the above the Tertiary sedimentary basins in Northeastern Greece remained thermally active until today. In conjunction with the ongoing extensional tectonic regime, extended hydrothermal systems were established in areas where lithology and active faults are properly present.

**b. The Tertiary basins of Northeastern Greece and the associated geothermal fields**

***The Loutros-Feres-Soufli basin (LFS)*** is one of the three subdepocenters of the Evros Delta (Alexandroupolis) Tertiary basin, composing the older sedimentary section in Southern Rhodopes [^4^](#_ENREF_4). It is located west of the Evros River being the widest and the most affected by subsidence eastwards [^12^](#_ENREF_12). The stratigraphic sequence is composed of a coarse-grained Lutecian basal clastic sequence, Priambonian sandstones, marls and conglomerates and an Oligocene sequence of marly and clayey sediments. The marine Paleogene-Eocene deposits are unconformably deposited upon the Mesozoic formations or to the west upon the gneisses, amphibolites and ophiolites of the Rhodope massif [^2^](#_ENREF_2). The thick Neogene and Quaternary sequence (max. thickness 1500 m) overlie uncomformably the Paleogene series of the basin and consists of clays, siltstones, sandstones and conglomerates.

A very intense volcanic activity in the region occurred during the Eocene and Oligocene, even down to the Early Miocene and it culminated during the Late Oligocene. Volcanic products outcropping on the margins of the basin consist of pyroclastics, interlayered with Oligocene sediments, rhyolitic ignimbrites, breccias, lava flows, dykes and domes. Volcanism ended in the Miocene with both acid and intermediate volcanic products [^12^](#_ENREF_12)^,^ [^14-16^](#_ENREF_14).

The geothermal interest in the wider area was initially recognized by the presence of two thermal springs, known from the Roman era, at Traianoupolis and Fylakto-Tychero with water temperatures 52°C and 36°C respectively.

**The *Xanthi-Komotini basin*** *(XK)* belongs to the Rhodope Massif. It is located on its southern margins and is bounded by the Circum-Rhodope Belt in the east and by the Avdira normal fault in the west, exhibiting a typical graben structure (fig. 1). The basin is delimited in the north by extended active faults with a dominant trend N70°, while southwards the up arching of the basement is characterized by step faults of Ν70° and N160°C directions. The most representative stratigraphic section found in the area of Iasmos consists of continental coarse-grained sandstones and conglomerates of Lutetian age overlain by Upper Eocene deep-water deposits capped by an erosional unconformity of Oligocene fluvial conglomerates. The eastern and central part of the basin is characterized by a relatively elevated heat flow regime and the subsequent presence of geothermal aquifers with low water temperatures ranging between 30°C and 45°C at depths 400-500 m.

On the contrary, on the western margin of the basin and most precisely in the area of Nea Kessani-Xanthi, we can reach one of the most significant and thoroughly explored low temperature geothermal field**s** in Northern Greece.

**The Nestos Delta basin** (ND) that extends over an about 450km^2^ area (onshore part), is forming the continental margin of the broader graben type basin of Prinos that includes the Nestos delta and the offshore extension between Thassos island and the main land around Kavala bay. It is bounded by two major faults striking N70◦ and N160◦, separated from the Xanthi-Komotini basin by the basement Avdira ridge (horst). The basement of the basin consists mainly of metamoprphic rocks (gneiss, amphibolites and marbles) of the Rhodope Massif and is characterized by high thermal gradient due to the granitic intrusions [^17^](#_ENREF_17). Sedimentation has begun with the deposition (0.7-3.0 km) of Miocene (Mid-Lower) sediments consisting of clastic/deltaic continental deposits, conglomerates with lignite intercalations and anhydrites alternating with thin layers of sandstones, clays and marls [^18^](#_ENREF_18). In the deeper parts of the basin a sequence of evaporitic marine sediments of Upper-Miocene age has been deposited on the clastic sediments. The evaporitic sequence is unconformably overlain by Plio-Quaternary sediments (up to 0.9 km), consisting mainly of deltaic sandstones, clays, marine and lacustrine sediments [^19^](#_ENREF_19).

Two significant low temperature geothermal fields have been recognized in the Nestos Delta basin, i.e. the geothermal fields of Erasmio and of Eratino (Fig.1).

***The Strymon River basin (SR)*** is an active post-orogenic graben structure shaped between the Mesozoic basement of the Serbo-Macedonian Massif (SRB) (amphibolites, magmatic gneiss and marbles) in the west, and the Rhodope Massif (marbles, gneiss and mica schists) intruded by the Vrondou granitic complex in the east. Due to various depositional paleoenvironments created during the Neogene-Quaternary that followed by lateral extension and transition, the stratigraphy of the basin is very complicated [^20^](#_ENREF_20). The older Miocene formations (basal conglomerates and breccias, alternations of clays, siltstones, sandstones, dark brown marls, lignite layers, petroliferous limestones), 700-800 m Pliocene sediments (layers of evaporates, conglomerates, travertines, marls, red clays, sandstones, siltstones, limestones, lignites) and 900-1000 m of Pleistocene sediments (alternations of shales, sands, clays, sandstones, marls, conglomerates and limestones) constitute the typical stratigraphic column of the basin, which at the center of the basin is evaluated to be close to 4000 m [^21^](#_ENREF_21)^,^ [^22^](#_ENREF_22). Pliocene to Quaternary volcanic rocks (rhyolites, tuffs) constitute the northern part of the Strymon basin. Their occurrence is strongly related to the extensional tectonics of the area.

The most relevant are the fields of: (a) Nigrita-Therma, (b) Sidirokastro, (3) Iraklia, (4) Agistro, (5) Achinos-Ivira and (6) Akropotamos. The principal characteristics of the geothermal fields and thermal springs outlined above are depicted in the table 1.

**Table S1**

Principal characteristics of the most relevant geothermal fields and thermal springs in North Eastern Greece (data is partly compiled from [^2^](#_ENREF_2)^,^ [^23-26^](#_ENREF_23))

| Tertiary basin (index) | Geothermal field - Spring | Reservoir roof depth (thickness) (m) | Reservoir formations of emerging thermal waters | Sample Code |
| --- | --- | --- | --- | --- |
|  |  |  |  |  |
| Loutros-Feres-Soufli (LFS) | Fylakto-Tychero- (GF) | 80 (200) | Rhyolites-Breccia | FΥL |
|  | Aristino_upper (GF) | 200 (20)  100-150 (20)**^1^** | Volcaniclastic  Altered dacitic lavas | ARS-7  ARS -2  ARS-12 |
|  | Aristino_lower (GF) | 100-250 (30)**^1^**  330-430 (30)**^1^** | Altered dacitic lavas-tuffs Ignimbrites | ARS-1,  ARS-3, ARS-4, ARS-5, ARS-6,  ARS-8, ARS-9,  ARS-10,  ARS-11 |
|  | Traianoupolis (TS) |  |  | TRA |
| Xanthi-Komotini (XK) | Nea kessani (GF) | 150-400 (50-250) | Conglomerates – Arcosic Sandstones | KES |
|  | Potamia (TS) |  |  | POT |
| Nestos River Delta(ND) | Erasmio_upper (GF) | 200 (60) | Sandstones | ERA-6 |
|  | Erasmio-lower (GF) | 290-400 (100) | Magmatic gneiss –Conglomerates | ERA-12 |
|  | Myrodato (GF) | 150-200 (50) | Arcosic Sandstones | MYR |
|  | Eratino-Chrysoupolis (GF) | 550-700 (40-100) | Limestones-Sandstones | ERC |
| Strymon River (SR) | Agistro (GF) | 50, 70-130 | Metamorphic basement | AGS |
|  | Sidirokastro (GF) | 10 (50), 100 (300) | Travertines, breccia | SID |
|  | Nigrita (GF) | 100-400 (20-65) | Conglomerates | NIG |
|  | Iraklia-Lithotopos (GF) | 270 (30) | Sandstones-conglomerates | IRA |
|  | Achinos-Ivira (GF) |  | Sands, sandstones, micro conglomerates | ACH |
|  | Akropotamos-up (GF) | 115 (85) | Pebbles, Sandstones | AKR-4 |
|  | Akropotamos-down (GF) | 240 (40)**^1^**  440 (80)**^1^** | Calcareous conglomerates-Sandstones | AKR-1  AKR-3 |
|  | Loutra Elefteron (TS) |  |  | ELF |
| RM* | Thermes (TS)***** |  |  | THE |

GF: Geothermal Field; TS: Thermal Spring; 1: Aquifers partially penetrated; *: Rhodope Massif


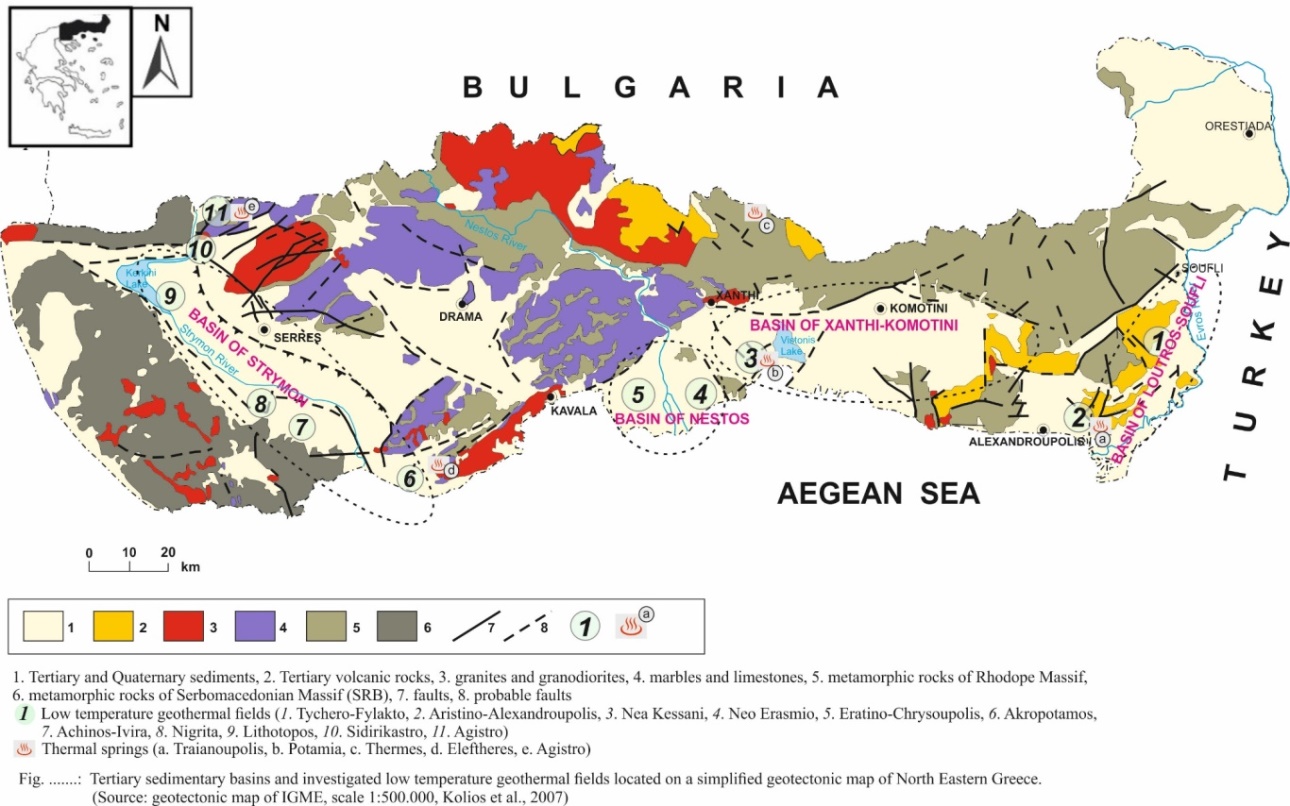


(1. Tertiary and Quatermary sediments, 2. Tertiary volcanic rocks, 3. granites and granodiorites, 4. marbles and limestones, 5. metamorphic rocks of Rhodope Massif, 6. metamorphic rocks of Serbomacedonian Massif (SRB), 7. faults, 8. probable faults.)
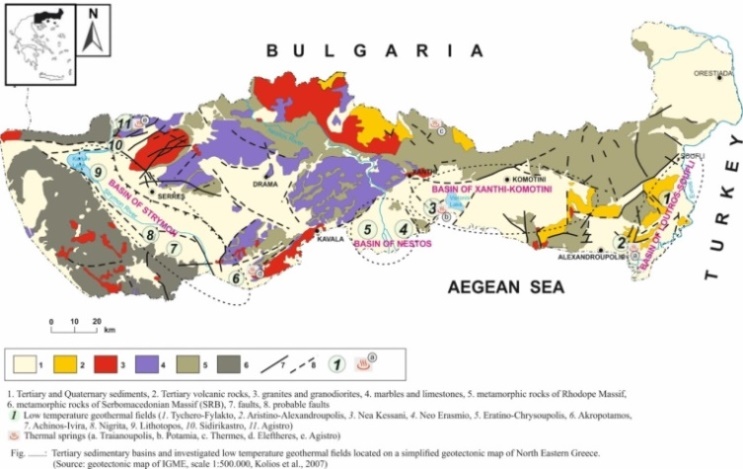
Low temperature geothermal fields (1. Tychero-Fylakto, 2. Aristino-Alexandroupolis, 3. Nea Kessani, 4. Myrodato-Neo Erasmio, 5. Eratino-Chrysoupolis, 6. Akropotamos, 7. Achinos-Ivira, 8. Nigrita, 9. Iraklia-Lithotopos, 10. Sidirokastro, 11. Agistro),
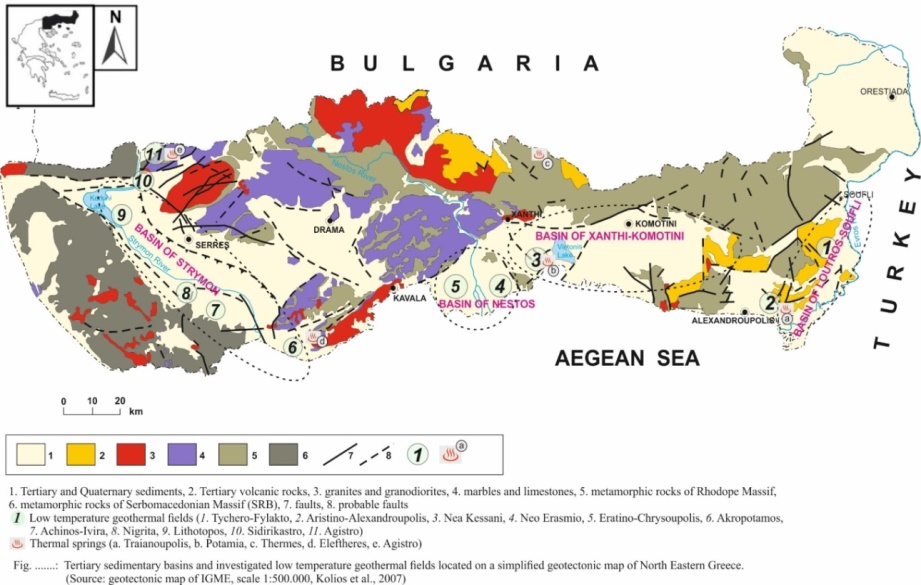
Thermal springs (a. Traianoupolis, b. Potamia, c. Thermes, d. Eleftheres, e. Agistro)

**Figure S1.** Tertiary sedimentary basins and investigated low temperature geothermal fields located on a simplified geotectonic map of North Eastern Greece. (scale 1:500.000, [^2^](#_ENREF_2)).

]

**Figure S2**. Ranges of δ^13^C_CO2_ and δ^13^C_TDIC_ values.

**Table S2.**  Chemical data and isotopic values

| Basin | Sample | pH | T | Cl^-^ | Br^-^ | HCO_3_^-^ | SO_4_^-2^ | SiO_2_ | Ca^2+^ | Mg^2+^ | Na^+^ | K^+^ | Li^+^ | B^+^ | δ^18^O | δ^2^H | δ^34^S SO_4_ | δ^18^O SO_4_ | δ^13^C  DIC | δ^13^C_CO2_ cal/ted |
| --- | --- | --- | --- | --- | --- | --- | --- | --- | --- | --- | --- | --- | --- | --- | --- | --- | --- | --- | --- | --- |
|  |  |  | ℃ | **(mg/l)** | | | | | | | | | | | **(‰)** | | | | | |
| SR |  |  |  |  |  |  |  |  |  |  |  |  |  |  |  |  |  |  |  |  |
|  | **Nigrita** |  |  |  |  |  |  |  |  |  |  |  |  |  |  |  |  |  |  |  |
|  | NIG-1 | 6.70 | 59 | 157.7 |  | 1970 | 105 | 71 | 122 | 95 | 529 | 82 | 1.45 | 3.5 | -8.1 | -58.5 |  |  |  |  |
|  | NIG-2 | 6.90 | 55 | 148.9 |  | 1670 | 127 | 71 | 52 | 83 | 517 | 78 |  | 2.8 | -8.1 | -58.5 |  |  |  |  |
|  | NIG-3 | 7.07 | 45 | 150.7 |  | 2360 | 142 | 50.5 | 236 | 105 | 533 | 98 | 0.80 | 2.5 | -8.1 | -58.5 |  |  |  |  |
|  | NIG-4 | 6.80 | 50 | 94.0 | 0.4 | 2110 | 140 | 100 | 190 | 140 | 580 | 80 | 0.70 | 3.4 | -8.8 | -57.3 | 20.8 | 6.8 | 8.2 | 0.2 |
|  | NIG-5 | 7.07 | 42 | 152.4 |  | 2020 | 139 | 56.2 | 130 | 109 | 524 | 90 | 0.80 | 3.4 | -8.1 | -58.5 |  |  |  |  |
|  | NIG-6 | 6.90 | 65 | 180.0 |  | 2100 | 128 | 102 | 140 | 100 | 610 | 80 | 1.00 | 3.3 | -8.8 | -57.3 | 20.3 | 6.6 |  |  |
|  | NIG-7 | 6.90 | 45 | 175.0 |  | 2150 | 130 | 102 | 145 | 10 | 605 | 82 | 1.00 | 3.3 | -8.4 | -58.0 | 20.2 | 6.7 |  |  |
|  | NIG-8 | 6.85 | 50 | 150.0 |  | 1960 | 105 | 102 | 120 | 9 | 530 | 80 | 1.10 | 3.6 | -8.6 | -57.0 | 20.8 | 6.5 |  |  |
|  | NIG-9 | 7.30 | 24 | 80.0 |  | 1080 | 135 | 100 | 185 | 142 | 585 | 84 | 0.20 | 2.6 | -8.8 | -60.0 |  |  |  |  |
|  | NIG-10 | 6.80 | 58 | 156.9 |  | 2060 | 116 | 82 | 140 | 91 | 537 | 92 | 0.90 |  |  |  |  |  |  |  |
|  | NIG-11 | 6.90 | 44 | 91.0 | 0.5 | 2100 | 120 | 102 | 150 | 150 | 530 | 70 | 0.20 | 3.3 | -8.4 | -56.1 | 20.9 | 6.3 | 8.1 | 0.1 |
|  | NIG-12 | 6.60 | 47 | 177.2 |  | 2260 | 130 | 85 | 148 | 117 | 621 | 78 | 0.90 | 3.4 |  |  |  |  |  |  |
|  | NIG-13 | 7.24 | 62 | 177.2 |  | 2170 | 128 | 85 | 140 | 102 | 598 | 78 | 0.90 | 3.4 |  |  |  |  |  |  |
|  | NIG-14 | - | 32 | 109.9 |  | 2190 | 456 |  | 27 | 3 | 414 | 9 |  | 2.4 |  |  |  |  |  |  |
|  | NIG-15 | 6.90 | 42 | 155.8 |  | 2200 | 128 | 91 | 131 | 102 | 523 | 80 | 1.00 | 3.4 |  |  |  |  |  |  |
|  | NIG-16 | 6.70 | 50 | 170.2 |  | 2110 | 126 | 94 | 138 | 113 | 575 | 78 | 0.80 | 3.2 |  |  |  |  |  |  |
|  | NIG-17 | 6.70 | 45 | 153.6 |  | 2076 | 130 | 59 | 137 | 109 | 531 | 95 | 1.10 | 3.5 |  |  |  |  |  |  |
|  | **Akropotamos** |  |  |  |  |  |  |  |  |  |  |  |  |  |  |  |  |  |  |  |
|  | AKR-1 | 7.20 | 90 | 762.0 |  | 1225 | 16 |  | 35 | 17 | 835 | 82 | 1.22 | 3.8 |  |  |  |  |  |  |
|  | AKR-2 | 7.30 | 84 | 2149.0 |  | 1269 | 151 |  | 390 | 18 | 1710 | 170 | 2.85 | 7.3 |  |  |  |  |  |  |
|  | AKR-3 | 6.85 | 84 | 2190.0 | 8 | 1150 | 245 | 70 | 128 | 14 | 1700 | 140 | 2.80 | 8.0 | -5.7 | -41.0 |  |  |  |  |
|  | AKR-4 | 7.40 | 45 | 630.0 | 2 | 980 | 190 |  | 41 | 20 | 730 | 15 | 0.20 | 2.5 |  |  |  |  |  |  |
|  | **Sidirokastro** |  |  |  |  |  |  |  |  |  |  |  |  |  |  |  |  |  |  |  |
|  | SID-1 | 6.78 | 41 | 70.9 |  | 799 | 209 | 34 | 138 | 23 | 225 | 36 | 0.44 | 3.4 | -8.8 | -61.0 |  |  | 1.7 | -6.3 |
|  | SID-2 | 6.60 | 55 | 53.2 |  | 900 | 204 | 39 | 152 | 23 | 238 | 37 | 0.50 | 2.3 | -8.8 | -61.0 |  |  | 1.6 | -6.4 |
|  | SID-3 | 6.70 | 42 | 51.4 |  | 1020 | 192 | 28 | 142 | 26 | 294 | 35 | 0.33 | 3.0 | -8.8 | -61.0 |  |  | 1.6 | -6.4 |
|  | SID-4 | 6.60 | 48 | 53.2 |  | 1100 | 192 | 28 | 169 | 26 | 268 | 43 | 0.35 | 3.0 | -8.8 | -61.0 |  |  | 1.6 | -6.4 |
|  | SID-5 | 6.40 | 42 | 55.1 |  | 1100 | 149 | 36 | 182 | 25 | 256 | 50 | 0.50 |  |  |  |  |  | 1.6 | -6.4 |
|  | SID-6 | 6.76 | 43 | 51.4 |  | 940 | 201 | 59 | 148 | 25 | 244 | 37 | 0.50 | 2.3 |  |  | 7.2 | 3.4 | 1.6 | -6.4 |
|  | SID-7 | 6.40 | 43 | 52.4 |  | 870 | 194 | 76 | 136 | 21 | 251 | 45 | 0.50 | 6.5 | -8.9 | -63.5 | 7.3 | 3.4 | 1.6 | -6.4 |
|  | SID-8 | 6.90 | 50 | 51.4 |  | 940 | 215 | 76 | 132 | 26 | 258 | 47 | 0.50 | 2.3 |  |  |  |  | 1.7 | -6.3 |
|  | SID-9 | 7.50 | 34 | 51.8 |  | 930 | 205 | 75 | 140 | 23 | 253 | 6 | 0.50 | 2.3 |  |  |  |  | 1.7 | -6.3 |
|  | **Agistro** |  |  |  |  |  |  |  |  |  |  |  |  |  |  |  |  |  |  |  |
|  | AGS-1 | 8.07 | 43 | 16.0 |  | 170 | 120 | 52 | 37 | 4 | 82 | 4 | 0.12 | 0.2 | -9.7 | -63.0 | 8.1 | 4.6 |  | -8.0 |
|  | AGS-2 | 7.80 | 43 | 14.2 |  | 170 | 143 | 60 | 32 | 5 | 94 | 4 | 0.10 | 0.2 | -9.8 | -65.0 | 8.4 | 4.6 |  | -8.0 |
|  | AGS-3 | 7.85 | 26 | 8.9 |  | 185 | 50 | 12 | 51 | 4 | 30 | 2 | 0.03 | 0.0 |  |  |  |  |  | -8.0 |
|  | AGS-4 | 8.00 | 29 | 17.7 |  | 160 | 202 | 55 | 40 | 5 | 109 | 5 | 0.15 | 0.3 | -9.7 | -64.5 |  | 4.6 |  | -8.0 |
|  | AGS-5 | 7.55 | 40 | 9.9 |  | 190 | 33 | 34 | 47 | 5 | 36 | 3 | 0.11 |  |  |  |  |  |  | -8.0 |
|  | **Iraklia-Lithotopos** |  |  |  |  |  |  |  |  |  |  |  |  |  |  |  |  |  |  |  |
|  | IRA-1 | 8.17 | 32 | 159.5 |  | 1800 | 48 | 16.5 | 16 | 29 | 713 | 45 | 0.15 | 4.0 |  |  |  |  |  |  |
|  | IRA-2 | 7.74 | 41 | 85.0 |  | 1040 | 160 | 62 | 16 | 9 | 505 | 14 | 0.17 | 3.6 |  |  |  |  |  |  |
|  | IRA-3 | 7.08 | 29 | 6.7 |  | 190 | 52 | 26 | 19 | 2 | 65 | 3 |  |  |  |  |  |  |  |  |
|  | IRA-4 | 7.40 | 31 | 56.7 |  | 610 | 145 | 60 | 18 | 8 | 292 | 8 |  | 1.2 |  |  |  |  |  |  |
|  | **Ivira-Achinos** |  |  |  |  |  |  |  |  |  |  |  |  |  |  |  |  |  |  |  |
|  | ACH-1 | 7.3 | 40 | 101.2 |  | 2510 | 102 | 35 | 18 | 6 | 1017 | 15 | 0.42 | 1.8 |  |  |  |  |  |  |
|  | ACH-2 | 8.05 | 27 | 51.0 |  | 430 | 84 | 21 | 33 | 20 | 160 | 4 | 0.07 | 0.4 |  |  |  |  |  |  |
|  | ACH-3 | 7.92 | 34 | 28.2 |  | 745 | 20 | 24 | 11 | 7 | 290 | 4 | 0.14 | 0.3 |  |  |  |  |  |  |
|  | ACH-4 | 7.40 | 32 | 23.0 |  | 490 | 46 | 26 | 3 | 1 | 235 | 4 | 0.02 | 0.1 |  |  |  |  |  |  |
|  | ACH-5 | 8.80 | 28 | 34.5 |  | 950 | 10 | 15 | 2 | 1 | 408 | 2 | 0.08 | 0.8 |  |  |  |  |  |  |
|  | ACH-6 | 7.72 | 29 | 33.5 |  | 920 | 166 | 18 | 14 | 10 | 418 | 5 | 0.21 | 0.7 |  |  |  |  |  |  |
|  | **Loutra Elefteron** |  |  |  |  |  |  |  |  |  |  |  |  |  |  |  |  |  |  |  |
|  | ELF-1 | 6.20 | 41 | 785.0 | 3 | 1150 | 25 | 45.8 | 210 | 19 | 650 | 54 | 1.20 | 2.7 | -5.7 | -41.0 |  |  | 9.0 | -1.0 |
|  | ELF-2 | 7.30 | 41 | 858.0 | 8 | 740 | 143 | 44.6 | 120 | 13 | 680 | 63 | 0.80 | 1.9 |  |  |  |  |  |  |
| ND |  |  |  |  |  |  |  |  |  |  |  |  |  |  |  |  |  |  |  |  |
|  | **Erasmio** |  |  |  |  |  |  |  |  |  |  |  |  |  |  |  |  |  |  |  |
|  | ERA-1 | 8.35 | 54 | 180.0 |  | 231.8 | 34 | 28 | 6 | 1 | 209 | 2 | 0.13 | 0.4 | -8.6 | -58.5 |  |  |  |  |
|  | ERA-2 | 7.87 | 30 | 370.0 |  | 356.2 | 154 | 18 | 24 | 9 | 402 | 11 |  | 1.5 | -8.5 | -58.0 |  |  |  | -8.0 |
|  | ERA-3 | 7.45 | 62 | 1830.0 |  | 59.8 | 269 | 24 | 401 | 12 | 844 | 14 | 0.43 | 1.1 |  |  |  |  |  |  |
|  | ERA-4 | 7.60 | 47 | 2090.0 |  | 189 | 217 | 30 | 313 | 31 | 1122 | 26 | 0.65 | 0.9 |  |  |  |  |  | -8.0 |
|  | ERA-5 | 8.35 | 55 | 99.0 |  | 242.8 | 59 |  | 3 | 1 | 178 | 2 |  |  |  |  |  |  |  |  |
|  | ERA-6 | 7.73 | 47 | 1630.0 |  | 132.9 | 165 |  | 228 | 7 | 900 | 26 |  |  |  |  |  |  |  |  |
|  | ERA-7 | 8.51 | 53 | 245.0 |  | 202.5 | 390 | 14 | 7 | 1 | 276 | 4 | 0.50 |  | -5.8 |  |  |  |  |  |
|  | ERA-8 | 6.45 | 42 | 3440.0 |  | 99.4 | 354 | 27 | 357 | 22 | 1947 | 59 | 1.20 | 3.3 |  |  |  |  |  |  |
|  | ERA-9 | 7.70 | 64 | 5570.0 |  | 42.7 | 340 | 70 | 601 | 15 | 3035 | 20 |  | 1.8 | -6.4 | -47.0 | 19.8 | 8.5 |  | -8.0 |
|  | ERA-10 | 7.29 | 60 | 5580.0 |  | 35.4 | 550 | 58 | 615 | 8 | 3000 | 220 |  |  |  |  |  |  |  | -8.0 |
|  | ERA-11 | 8.50 | 67 | 210.0 |  | 216 | 36 | 37 | 3 | 1 | 234 | 4 | 0.14 | 0.2 |  |  |  |  |  |  |
|  | ERA-12 | 8.50 | 68 | 178.0 |  | 225.8 | 34 | 34 | 4 | 2 | 212 | 4 | 0.23 | 0.4 |  |  |  |  |  | -19.7 |
|  | **Eratino-Chrysoupolis** |  |  |  |  |  |  |  |  |  |  |  |  |  |  |  |  |  |  |  |
|  | ERC-1 | 8.15 | 25 | 337.0 |  | 420 | 60 | 25 | 2 | 2 | 386 | 16 |  | 0.9 |  |  |  |  |  |  |
|  | ERC-2 | 7.95 | 27 | 375.8 |  | 445 | 46 | 30 | 18 | 9 | 395 | 7 |  | 0.5 | -8.5 | -58.0 |  |  |  |  |
|  | ERC-3 | 6.90 | 21 | 5033.7 |  | 304 | 706 | 7 | 249 | 326 | 2713 | 137 | 0.80 | 0.8 |  |  | 20.2 | 8.1 |  |  |
|  | ERC-4 | 7.30 | 21 | 5264.1 | 19 | 107 | 6 | 25 | 57 | 44 | 2690 | 43 | 0.80 | 1.4 | -6.5 | -48.0 | 17.1 | 8.5 | -10.5 | -18.5 |
|  | ERC-5 | 7.08 | 65 | 8510.4 |  | 104 | 963 | 39 | 862 | 180 | 4500 | 68 |  | 10.1 | -5.8 | -42.5 |  |  | -10.4 | -18.4 |
|  | ERC-6 | 7.45 | 55 | 6879.2 |  | 140 | 890 | 32 | 681 | 170 | 3800 | 67 |  | 6.6 |  |  |  |  | -9.6 | -17.6 |
|  | ERC-7 |  | 47 | 230.0 |  | 350 | 680 | 30 | 2 | 2 | 320 | 14 |  |  |  |  |  |  | -11.4 | -19.4 |
|  | ERC-8 |  | 63 | 5800.0 | 19 | 1600 | 480 | 30 | 600 | 140 | 2900 | 60 | 0.00 |  | -6.4 | -44.8 |  |  |  |  |
|  | **Myrodato** |  |  |  |  |  |  |  |  |  |  |  |  |  |  |  |  |  |  |  |
|  | MYR-1 | 8.60 | 51 | 2070.0 |  | 650 | 680 |  | 170 | 17 | 1530 | 120 |  | 4.2 | -6.3 | -49.0 | 7.9 | 3.4 |  |  |
| LFS |  |  |  |  |  |  |  |  |  |  |  |  |  |  |  |  |  |  |  |  |
|  | **Aristino** |  |  |  |  |  |  |  |  |  |  |  |  |  |  |  |  |  |  |  |
|  | ARS-1 | 7.00 | 86 | 4610.0 |  | 190 | 725 | 85 | 325 | 5 | 2770 | 140 | 3.00 | 7.0 | -6.5 | -49.0 |  |  |  | -8.0 |
|  | ARS-2 | 7.80 | 43 | 145.0 |  | 440 | 1500 | 34 | 95 | 10 | 1040 | 20 |  | 3.2 |  |  |  |  |  | -8.0 |
|  | ARS-3 | 7.95 | 79 | 3748.0 |  | 180 | 775 | 63 | 380 | 14 | 3200 | 130 |  | 5.7 | -6.5 |  |  |  |  | -8.0 |
|  | ARS-4 | 7.40 | 85 | 2252.0 |  | 240 | 1000 |  | 235 | 15 | 1780 | 135 |  |  | -6.4 | -48.5 |  |  |  | -8.0 |
|  | ARS-4 | 7.04 | 93 | 4960.0 |  | 215 | 800 | 85 | 560 | 20 | 3080 | 150 | 6.90 | 7.0 | -6.5 | -48.0 | 20.2 | 6.0 |  | -8.0 |
|  | ARS-5 | 7.44 | 72 | 4255.2 |  | 125 | 236 | 64 | 341 | 24 | 2000 | 100 | 7.00 | 7.0 | -6.5 | -48.5 |  |  |  | -8.0 |
|  | ARS-6 | 7.72 | 52 | 5283.5 |  | 170 | 713 | 50 | 521 | 34 | 2760 | 100 | 6.90 | 6.5 | -6.5 | -48.0 |  |  |  |  |
|  | ARS-7 | 7.27 | 31 | 4982.1 |  | 306 | 675 | 15 | 472 | 23 | 2600 | 100 | 7.80 | 11.3 | -6.6 | -48.9 |  |  |  | -8.0 |
|  | ARS-8 | 7.89 | 87 | 5095.6 |  | 870 | 510 | 41 | 353 | 29 | 3700 | 57 |  | 10.0 | -6.5 | -50.0 |  |  |  | -8.0 |
|  | ARS-9 | 7.58 | 89 | 5655.9 |  | 85 | 750 | 23 | 517 | 12 | 3400 | 104 | 7.10 | 7.0 |  |  |  |  |  |  |
|  | ARS-10 | 7.72 | 60 | 4609.8 |  | 104 | 665 | 25 | 329 | 10 | 2950 | 23 |  | 5.4 |  |  |  |  |  | -8.0 |
|  | ARS-11 | 7.91 | 64 | 5212.6 |  | 70 | 710 |  | 392 | 15 | 3300 | 30 | 7.00 | 8.8 |  |  |  |  |  |  |
|  | ARS-12 | 7.95 | 33 | 3840.0 |  | 210 | 625 |  | 334 | 10 | 2500 | 100 |  | 9.0 | -6.4 |  |  |  |  | -8.0 |
|  | **Traianoupoli** |  |  |  |  |  |  |  |  |  |  |  |  |  |  |  |  |  |  |  |
|  | TRA-1 |  | 51 | 4800.0 | 17 | 165 | 480 | 42 | 550 | 1360 | 2370 | 120 | 5.30 | 5.1 |  |  |  |  | -7.6 | -15.6 |
|  | TRA-2 |  | 50 | 3750.0 |  | 160 | 775 |  | 180 | 14 | 3200 | 130 | 3.00 | 6.0 | -6.5 | -47.0 |  | 3.1 | -7.4 | -15.4 |
|  | **Fylakto** |  |  |  |  |  |  |  |  |  |  |  |  |  |  |  |  |  |  |  |
|  | FYL | 8.00 | 37 | 103.0 |  | 96 | 81 |  | 70 | 1 | 120 | 3 | 0.62 |  |  |  |  |  |  |  |
| XK |  |  |  |  |  |  |  |  |  |  |  |  |  |  |  |  |  |  |  |  |
|  | **Nea Kessani** |  |  |  |  |  |  |  |  |  |  |  |  |  |  |  |  |  |  |  |
|  | KES-1 | 6.70 | 46 | 1675.5 |  | 1870 | 229 | 18 | 228 | 30 | 1483 | 156 |  | 4.8 |  |  |  |  |  |  |
|  | KES-2 | 6.80 | 72 | 1680.0 | 7.4 | 1522 | 250 | 64 | 330 | 25 | 1490 | 130 | 2.70 | 4.0 | -6.2 | -50.6 | 19.6 | 2.8 | 7.9 | -0.1 |
|  | KES-3 | 7.20 | 76 | 1595.7 |  | 1550 | 233 | 54 | 128 | 20 | 1455 | 147 |  | 4.8 | -6.2 | -49.0 |  |  |  |  |
|  | KES-4 | 6.70 | 64 | 1595.7 |  | 1550 | 233 | 48 | 134 | 16 | 1455 | 147 |  | 4.8 |  |  |  |  |  |  |
|  | KES-5 | 6.70 | 65 | 1825.0 | 7.3 | 1550 | 220 | 56 | 210 | 8 | 1550 | 130 | 1.40 | 4.2 | -6.4 | -50.2 | 18.4 | 3.6 | 8 | 0.0 |
|  | KES-6 | 8.00 | 30 | 99.3 |  | 280 | 125 | 30 | 4 | 1 | 175 | 4 |  | 0.1 | -8.2 | -53.0 |  |  |  |  |
|  | KES-7 | 7.60 | 32 | 76.2 |  | 265 | 64 | 11 | 37 | 5 | 107 | 2 |  | 0.1 | -8.2 | -53.0 |  |  |  |  |
|  | KES-8 | 6.95 | 67 | 1702.1 | 7.2 | 1720 | 226 | 44 | 135 | 25 | 1481 | 196 | 4.00 | 5.0 |  |  |  |  |  |  |
|  | KES-9 | 6.81 | 72 | 1613.5 |  | 1570 | 230 | 49 | 135 | 25 | 1480 | 195 | 1.40 | 5.0 | -6.1 | -49.0 |  |  |  |  |
|  | KES-10 | 7.37 | 78 | 1764.2 |  | 1560 | 227 | 46 | 130 | 35 | 1546 | 131 | 1.60 | 6.0 |  |  |  |  |  |  |
|  | KES-11 | 6.60 | 39 | 1539.0 | 7.1 | 1890 | 216 | 15 | 228 | 48 | 1380 | 136 | 1.20 | 4.3 |  |  |  |  |  |  |
|  | KES-12 | 7.00 | 54 | 1613.5 |  | 1550 | 237 | 53 | 133 | 18 | 1471 | 156 |  | 4.0 |  |  |  |  |  |  |
|  | KES-13 | 7.03 | 53 | 1613.5 |  | 1500 | 248 | 53 | 132 | 19 | 1472 | 156 |  | 4.0 |  |  |  |  |  |  |
|  | KES-14 | 7.08 | 52 | 1613.5 | 7 | 1570 | 254 | 52 | 138 | 16 | 1472 | 156 |  | 3.5 |  |  |  |  |  |  |
|  | KES-15 |  | - |  |  |  |  |  |  |  |  |  |  |  | -8.5 | -53.0 |  |  |  |  |
|  | KES-16 |  | - |  |  |  |  |  |  |  |  |  |  |  | -7.5 | -48.0 |  |  |  |  |
|  | **Potamia** |  |  |  |  |  |  |  |  |  |  |  |  |  |  |  |  |  |  |  |
|  | POT | 7.60 | 51 | 1650.0 |  | 1200 | 240 |  | 150 | 19 | 1430 | 150 | 1.70 | 5.8 | -6.4 | -49.0 | 20.1 | 7.4 | 3.1 | -4.9 |
| MR |  |  |  |  |  |  |  |  |  |  |  |  |  |  |  |  |  |  |  |  |
|  | **Thermes** |  |  |  |  |  |  |  |  |  |  |  |  |  |  |  |  |  |  |  |
|  | THE | 7.50 | 51 | 50.0 |  | 660 | 90 |  | 155 | 11 | 150 | 20 |  | 0.2 | -8.0 | -55.0 | 9.1 | 4.4 | 1.5 | -6.5 |
|  |  |  |  |  |  |  |  |  |  |  |  |  |  |  |  |  |  |  |  |  |
| SW | **Seawater** |  | 15 | 21000 | 70 | 160 | 3000 |  |  |  |  |  | 0.20 | 5.0 | 1.0 | 3.0 | 20.0 | 9.5 |  |  |

**Table S3**. Seawater contributions (%) in selected thermal waters calculated from the mass equation of Cl^-^ and Br^-^ concentrations. MSW: mixing with sea water.

| **Location** | **Sample** | **MSW % (Cl^-^)** | **MSW % (Br^-^)** | **MSW % (^18^O)** | **MSW % (^2^H)** |
| --- | --- | --- | --- | --- | --- |
| LFS basin | ARS-1 | 22 |  | 21 | 15 |
|  | ARS-3 | 18 |  | 21 |  |
|  | ARS-4 | 24 |  | 21 | 16 |
|  | ARS-5 | 23 | 24 | 21 | 16 |
|  | ARS-6 | 18 |  | 21 | 16 |
|  | ARS-7 | 21 |  | 20 | 15 |
|  | ARS-8 | 26 |  | 21 | 13 |
|  | ARS-9 | 24 |  |  |  |
|  | ARS-10 | 25 |  |  |  |
|  | ARS-11 | 22 |  |  |  |
|  | TRA-2 | 18 |  | 21 | 18 |
| ND basin | ERC-1 | 16 | 28 | 24 | 25 |
|  | ERC-2 | 18 |  |  | 25 |
|  | ERC-3 | 24 |  |  | 17 |
|  | ERC-4 | 25 | 27 | 21 | 14 |
|  | ERC-5 | 40 |  | 28 | 25 |
|  | ERC-6 | 32 |  |  |  |
|  | ERC-8 | 27 | 27 | 22 | 22 |
|  | ERA-7 | 12 |  |  |  |
|  | ERA-8 | 16 |  |  |  |
|  | ERA-9 | 26 |  | 23 | 19 |
|  | ERA-10 | 26 |  |  |  |
| SR basin | AKR-2 | 10 |  |  |  |
|  | AKR3 | 10 | 11 |  |  |

References

1. P.P.C. 42-53 (Public Petroleum Corporation, Athens, 1988).

2. Kolios, N., Fytikas, M., Arvanitis, A., Andritsos, N. & Koutsinos, S. in Proceedings European Geothermal Congress (2007).

3. Stampfli, G.M. & Borel, G.D. in The TRANSMED Atlas. The Mediterranean region from crust to mantle 53-80 (Springer, 2004).

4. Caracciolo, L. et al. The Tertiary Thrace basins of SE Bulgaria and NE Greece: a review of petrological and mineralogical data of sedimentary sequences. *Vulcanologica* **25**, 21-41 (2015).

5. Fytikas, M. et al. Tertiary to Quaternary evolution of volcanism in the Aegean region. *Geological Society, London, Special Publications* **17**, 687-699 (1984).

6. Burchfiel, C.B., Nakov, R., Tzankov, T. & Royden, L.H. Cenozoic extension in Bulgaria and northern Greece: the northern part of the Aegean extensional regime. *Geological Society, London, Special Publications* **173**, 325-352 (2000).

7. Marchev, P. et al. Compositional diversity of Eocene–Oligocene basaltic magmatism in the Eastern Rhodopes, SE Bulgaria: implications for genesis and tectonic setting. *Tectonophysics* **393**, 301-328 (2004).

8. Marchev, P. et al. Adakitic magmatism in post-collisional setting: an example from the Early–Middle Eocene Magmatic Belt in Southern Bulgaria and Northern Greece. *Lithos* **180**, 159-180 (2013).

9. Bonev, N. & Beccaletto, L. From syn-to post-orogenic Tertiary extension in the north Aegean region: constraints on the kinematics in the eastern Rhodope–Thrace, Bulgaria–Greece and the Biga Peninsula, NW Turkey. *Geological Society, London, Special Publications* **291**, 113-142 (2007).

10. Bonev, N. & Dilek, Y. Geochemistry and tectonic significance of proto‐ophiolitic metamafic units from the Serbo‐Macedonian and western Rhodope massifs (Bulgaria‐Greece). *International Geology Review* **52**, 298-335 (2010).

11. Bonev, N., Moritz, R., Marton, I., Chiaradia, M. & Marchev, P. Geochemistry, tectonics, and crustal evolution of basement rocks in the Eastern Rhodope Massif, Bulgaria. *International Geology Review* **52**, 269-297 (2010).

12. Christofides, G., Pécskay, Z., THERIADIS, G.E., SOLDATOS, T.Y. & Koroneos, A. The Tertiary Evros volcanic rocks (Thrace, northeastern Greece): petrology and K/Ar geochronology. *Geologica Carpathica* **55** (2004).

13. Kolios, N. (ed. I.G.M.E.) (Thessaloniki 2001).

14. Elefteriadis, G., Pe-Piper, G., Christofides, G., Soldatos, T. & Esson, J. K-Ar dating of the Samothraki volcanic rocks, Thrace, North-Eastern Aegean (Greece). *Bull. Soc. Geol. Greece* **30**, 205-212 (1994).

15. Innocenti, F. et al. The geology and geodynamic significance of the island of Limnos, North Aegean sea, Greece. *Neues Jahrbuch für Geologie und Paläontologie-Monatshefte*, 661-691 (1994).

16. Vlahou, M., Christofides, G., Eleftheriadis, G., Pinarelli, L. & Kassoli-Fournaraki, A. Major, trace element and SR-isotope characterization of the Samothraki tertiary volcanic rocks, NE Aegean. *Bulletin of the Geological Society of Greece* **34**, 995-1002 (2001).

17. Kolios, N., Koutsinos, S., Arvanitis, A. & Karydakis, G. in Proc. World Geothermal Congress Antalya, Turkey 24-29 (2005).

18. Proedrou, P. (Athens, 1979).

19. Karytsas, C. (ETD/89-6, Pikermi, Greece, 1990).

20. Syrides, G. Neogene marine cycles in Strymon basin, Macedonia, Greece. *Geol. Soc. Greece, Sp. Publ.* **9**, 217-225 (2000).

21. Mendrinos, D., Choropanitis, I., Polyzou, O. & Karytsas, C. Exploring for geothermal resources in Greece. *Geothermics* **39**, 124-137 (2010).

22. Lalechos, N. Correlations and observations in molassic sediments in onshore and offshore areas of northern Greece. *Mineral Wealth* **1986**, 7-34 (1986).

23. Karydakis, G., Arvanitis, A., Andritsos, N. & Fytikas, M. in Proceedings world geothermal congress. Turkey (2005).

24. Kolios, N. (Thesis, Un. of Athens, 1993).

25. Dotsika, E., Potoukis, D. & Dalampakis, P. in 4th hydrogeological congress 352-365. (Thessaloniki, 1997).

26. Poutoukis, D. & Dotsika, E. Contribution of isotope hydrology and hydrogeochemistry in the study of the geothermal filed of Aristino area, Evros. *Bull. Soc. Geol. Greece* **XXXII**, 69-79 (1998).
